# Acute social and physical stress interact to influence social behavior: The role of social anxiety

**DOI:** 10.1371/journal.pone.0204665

**Published:** 2018-10-25

**Authors:** Bernadette von Dawans, Amalie Trueg, Clemens Kirschbaum, Urs Fischbacher, Markus Heinrichs

**Affiliations:** 1 Department of Psychology, Laboratory for Biological and Personality Psychology, University of Freiburg, Freiburg, Germany; 2 Department of Psychology, Biological and Clinical Psychology, University of Trier, Trier, Germany; 3 Department of Psychology, Biological Psychology, Technical University of Dresden, Dresden, Germany; 4 Department of Economics, Applied Research in Economics, University of Konstanz, Konstanz, Germany; 5 Thurgau Institute of Economics, Kreuzlingen, Switzerland; Technion Israel Institute of Technology, ISRAEL

## Abstract

Stress is proven to have detrimental effects on physical and mental health. Due to different tasks and study designs, the direct consequences of acute stress have been found to be wide-reaching: while some studies report prosocial effects, others report increases in antisocial behavior, still others report no effect. To control for specific effects of different stressors and to consider the role of social anxiety in stress-related social behavior, we investigated the effects of social versus physical stress on behavior in male participants possessing different levels of social anxiety. In a randomized, controlled two by two design we investigated the impact of social and physical stress on behavior in healthy young men. We found significant influences on various subjective increases in stress by physical and social stress, but no interaction effect. Cortisol was significantly increased by physical stress, and the heart rate was modulated by physical and social stress as well as their combination. Social anxiety modulated the subjective stress response but not the cortisol or heart rate response. With respect to behavior, our results show that social and physical stress interacted to modulate trust, trustworthiness, and sharing. While social stress and physical stress alone reduced prosocial behavior, a combination of the two stressor modalities could restore prosociality. Social stress alone reduced nonsocial risk behavior regardless of physical stress. Social anxiety was associated with higher subjective stress responses and higher levels of trust. As a consequence, future studies will need to investigate further various stressors and clarify their effects on social behavior in health and social anxiety disorders.

## Introduction

Chronic stress reduces wellbeing, exacerbates mental disorders, and can be a significant risk factor for several diseases [1–3]. The stress response comprises several psycho-biological levels or parameters. These psycho-physiological adaptations help the organism adjust to environmental demands that may require increases in blood sugar or other metabolic alterations. These metabolic changes ensure the maintenance of homeostasis in the body [1]. The hypothalamus pituitary axis (HPA) with salivary cortisol as its most prominent marker, the sympathetic adrenomedullary system (SAM) (e.g heart rate), as well as the subjective psychological stress response measured via questionnaire represent important branches and variables of the stress response that should be captured in state-of-the-art research. While these axes are characterized by various complex feedback mechanisms and interactions, they may also respond quite independently, meaning that increases in one stress system do not necessarily lead to the same increases in all other stress systems—evident in the largely weak correlations between psychological and physiological stress responses [4]. It is the type of stressor that seems to modulate the three stress-response dimensions, with social evaluation being the key feature driving the cortisol stress response [5,6]. Moreover, several ‘features’ of the subject itself mediate the effectiveness of stressors regarding their varying levels of stress response. Gender, the menstrual cycle phase, or oral contraceptives, age or body weight [7,8] but also psychological variables such as personality traits or different psychopathological symptoms have exhibited an influence on all three stress levels [9–13]. One important situational variable affecting the stress response is ‘social evaluation’ [5]. This effect is dependent on one`s subjective appraisal and personal experiences, and is closely associated with social anxiety. Social evaluation is the feature of the TSST [14] or the Socially Evaluated Cold Pressor Test (SECPT) [6] that characterizes these paradigms as ‘social’ stress paradigms compared to non-social, physical stress paradigms like the Cold Pressor Test (CPT) [15], which lacks a social component. Manifold concepts from different decades and fields of sciences reveal the diversity of the concept ‘stress’ [16–20]. They attempt to provide a framework of mechanistic physiological action and behavioral consequences of stress. Although the fight-or-flight concept [21,22] represents for acute stress the dominant theoretical framework in both animal and human stress research, recent studies provide evidence that acute stressors can also lead to an increase in prosocial behavior [23–28]. They are derived from a theory called the tend-and-befriend concept [29,30]. The latter introduced the neuropeptide oxytocin and opioids as being involved in stress regulation and in the behavioral consequences of the stress response that may be affiliatory. These concepts highlight the diversity of behavioral findings in stress research, and reveal the ambiguity of research on the effects of stress on behavior. There is evidence supporting the fight-or-flight response to stress: e.g. Steinbeis and colleagues [31] report stressed participants as being less trusting. There is evidence that stress leads to less antisocial risk aversion in healthy subjects [32], and that stress reduced donations to a charitable organization [33]. With regard to moral decision-making, one study revealed no group differences between the stress and control group, but documented a positive correlation between the cortisol responses and egoistic decision-making in emotional dilemmas [34]. On the other hand, there are studies supporting the tend-and-befriend reaction to stress entailing higher levels of trust, trustworthiness, or sharing after acute stress [27], studies linking stress reactivity to better social cognition (already implying gender differences) [25,26,28], and studies indicating an association among stress induction, cortisol increase, and prosocial or affiliatory behaviors [23,35]. In the context of moral decision-making, a recent study reported higher levels of altruistic decisions in the stress than the control group [36].

Whether acute stress leads to prosocial, antisocial, or risky behavior depends upon various situational aspects, the kind of stressor, and the individual [24,33,37,38]. Several aspects of the study design (e.g. situational factors, the time gap between stressor and dependent variables) are known to be relevant [33,38] but the kind of stressor (social vs. nonsocial) has not been investigated yet. Individual differences also contribute to variations in stress reactivity per se [39] and may also modulate the behavioral consequences of stress; in particular, social anxiety may be a key factor in understanding the behavioral responses to acute stress exposure [40–45]. As social behavior itself is modulated by the social-anxiety trait [43,46,47], and the fear of social evaluation is the key problem associated with social anxiety, we set out to disentangle the effects of standardized physical versus social stress and the impact of social anxiety on social decision-making. Are the effects of acute stress on social decision-making mediated by the social aspects (social evaluation) of acute stress? Does social anxiety influence the effects of stress on behavior? We hypothesized that only social stress would increase prosocial behavior, and that this effect would be moderated by the level of social anxiety, i.e. participants presenting lower levels of social anxiety should exhibit increased prosocial behavior following acute psychosocial stress exposure, while participants with higher levels of social anxiety would not reveal an increase in prosocial behavior.

## Methods

### Participants

Online and telephone interviews were used to exclude potential participants who were not fluent in the German language, had acute or chronic psychiatric or medical illness, were taking prescription medication, worked the night shift, abused drugs or alcohol, or smoked more than five cigarettes per day. Potential participants completed the Social Interaction Anxiety Scale (SIAS) [48] online prior to the experiment and were stratified into four groups to ensure a normal distribution of social anxiety symptoms in each of four experimental groups. Depending on their score in the SIAS, participants were included as low (score of 0–23) or highly (score of > 23) socially anxious, in order to ensure an equal distribution of social anxiety among the experimental groups. An SIAS-score of 24 was chosen as an optimal point for differentiation in high and low social anxiety, based on Stangier et al. [28]. High and low socially anxious participants were then randomly assigned to the four experimental groups: warm water test (WWT: no social stress and no physical stress, N = 31), socially-evaluated warm water test (SEWWT: social stress but no physical stress, N = 34), cold pressor test (CPT: no social stress but physical stress, N = 44), and socially evaluated cold pressor test (SECPT: social stress and physical stress, N = 47). We decided to test more participants in the physical stress conditions according to the reported non-responder rates [6]. Moreover, participants needed to be naïve to the stress protocols employed (see *Physical and social stress induction*) and similar stress paradigms (Trier Social Stress Test (TSST) and the TSST-G (group version); [14,49]. Participants could not be students of psychology or economics and had to be unfamiliar with other participants and the experimenters. An exclusively male sample was recruited in order to circumvent the previously-reported modulatory effects of female menstrual cycle on the psychobiological stress response [7] as well as the effects of gender in social interaction paradigms [50,51]. As we regard the cortisol stress response as a prerequisite reflecting a robust physical stress response, we only included participants from our cold pressor and socially evaluated cold pressor task who revealed a minimum increase of 2 nmol/l (for details see [6]). Four out of the originally 156 healthy men between 18 and 40 years of age were outliers (+/- 2 SD) in social anxiety symptoms and therefore excluded from our analyses. Hence, those participants we screened and randomized to the four experimental groups are called *target participants*. They received 20€ for participating in the study and additional earnings from the social interaction task (mean = 5.59€, SD = 0.80€). The study was approved by the institutional review board of the University of Freiburg, Germany. A second group of participants was recruited as *interaction partners* for the target participants. This second group was involved only in the interaction games.

### Psychometric measures

We used the German version of the Social Interaction Anxiety Scale (SIAS) [48,52] to assess individual levels of social anxiety. This scale has 20 items rated on a 4 point Likert scale from 0 (not at all) to four (extremely). The items refer to situations feared by those with high social anxiety, eg, “I have difficulty making eye contact with others” or “I am nervous mixing with people I don’t know well”. The scale has good internal consistency with Cronbachs α between .88 and .93 and a sum score between 0 and 80. The German version of the Beck Depression Inventory (BDI) [53] was used to assess depressive symptoms in the participants with Cronbachs α ranging between 0.89 and 0.93 [54]. This standard scale assesses the cognitive affective and somatic aspects of depressive symptomatology on a 21-item scale. With the *Wortschatztest* with Cronbachs α = 0.94 we measured a proxy of verbal intelligence (verbal IQ) in our target participants [55]. The questionnaires were filled out online before the experiment via the platform Qualtrics.

### Physical and social stress induction

To compare biobehavioral responses to socio-evaluative and physical stress, we used standardized laboratory paradigms. Social evaluation (control condition: no evaluation) has been repeatedly shown to induce psycho-physiological stress responses in humans and is one core feature of the Trier Social Stress Test [14,49]. Cold water at a temperature of 0–4°C (control condition: warm water) has been used as a physical stressor since the 1970s [15]. Our study design thus consisted of four conditions: warm water test (WWT: no social and no physical stress), socially-evaluated warm water test (SEWWT: social evaluation but no physical stress), cold pressor test (CPT: no social stress but physical stress) and socially-evaluated cold pressor test (SECPT: social stress and physical stress). Schwabe and colleagues [6] designed these conditions recently adapted to accommodate the group setting [56]. We tested participants in groups of four to six individuals. We chose this group size in order to investigate social interaction paradigms that we already tested with the TSST-G procedure originally set up for groups of six participants [6,56]. We decided to test groups containing four to six people since the group size itself might affect the stress response and social interaction [49,57]. Participants were separated by mobile walls and were not able to interact or evaluate each other. In detail, in the physical stress condition participants were instructed to immerse their non-dominant hand in 0–4°C water (warm water condition: 37–40°C). They were told to keep their hand in the water as long as possible. After 3 minutes they were instructed to remove their hand. In the social-evaluation condition, participants were told they would be videotaped and that their facial expressions would be analyzed in these recordings; the two experimenters wore white coats and observed the participants constantly (no social evaluation: the experimenters wore no white coats, did not observe the participants, and there were no video cameras).

### Social and nonsocial decision paradigms

**Fig 1:**
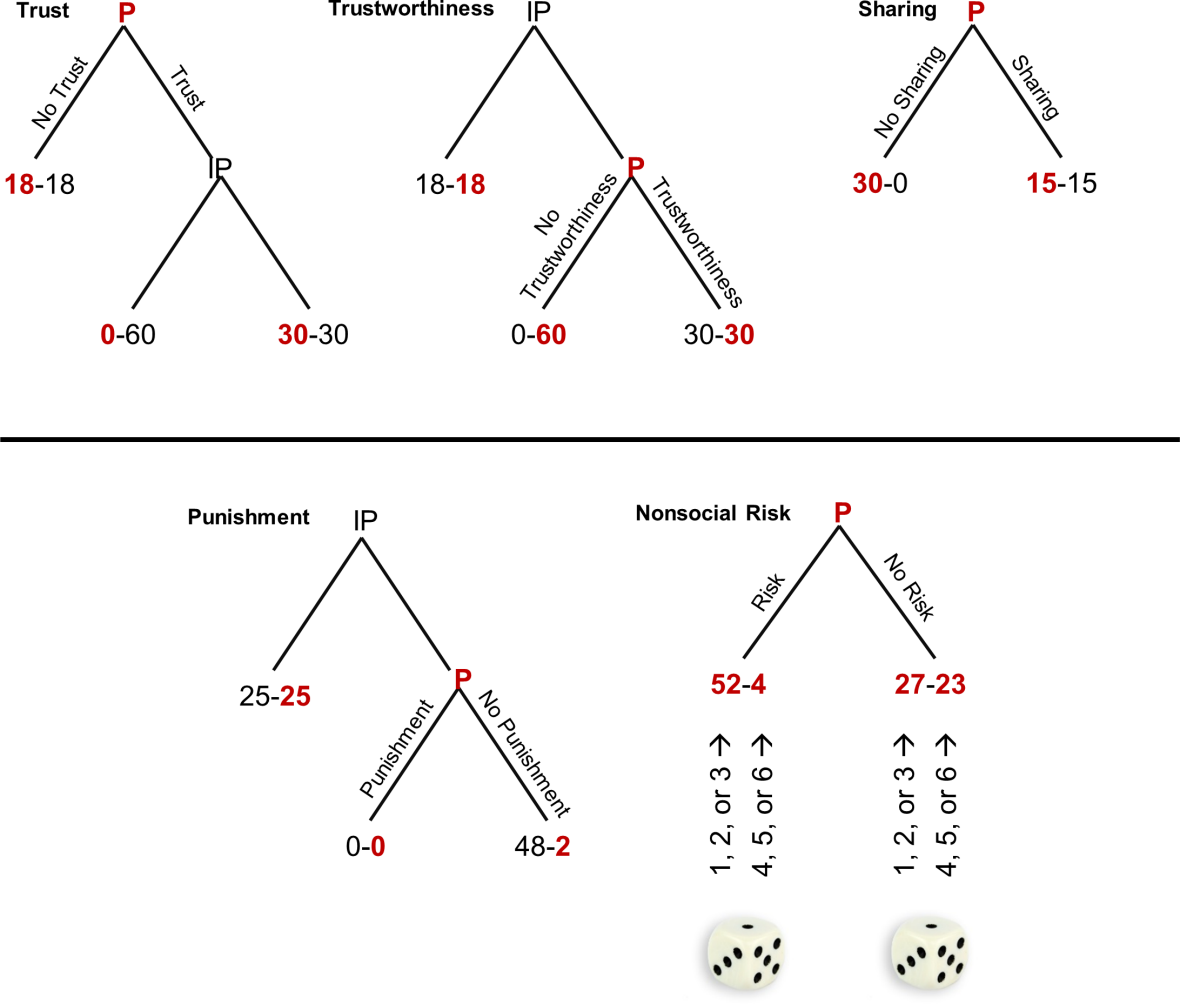
Examples for each game. The target participant is presented by a red P, participant’s interaction partner is represented by black IP, respectively (interaction partners were not in any of the social or physical stress conditions). The red value indicates the outcome for the target participant, the black value the outcome for the interaction partner. In the nonsocial risk game a die was rolled to determine the outcome.

To disentangle the effects of social versus physical stress on social behavior, we used a social decision-making task previously described in the context of stress exposure [27]. A set of decisions was used to study prosocial behavior (trust, trustworthiness, sharing; four decisions each), aggressive behavior (punishment; four decisions), and nonsocial risk behavior (8 decisions). Fig 1 shows one variant of each paradigm (see S1 Fig, supporting information (SI) for detailed parameters). Target participants interacted anonymously with interaction partners that did not take part in the social or physical stress conditions and were invited to the lab separately. Each decision was a binary choice (eg, trust vs. no trust, trustworthiness vs. no trustworthiness).

The *trust game* and *trustworthiness game* were sequential two player games. The player with the first move could choose to trust or not to trust. If the first player trusted, a higher number of points could be gained depending on whether the second player was trustworthy or not. The subjects played four variants of the game as player with the first move (trust) and four variants thereof as player with the second move (trustworthiness). The second player had to decide whether to be trustworthy or not before he being informed about the first mover choice, which is called the strategy method.

The *punishment game* was again a sequential two-player game. The interaction partner always had the first move, and he could decide how to distribute 50 MU. He could either choose a fair or a given unfair distribution. If he chose the fair offer, there was no further choice. But if he chose the unfair offer, the target participant could either accept the offer or punish the interaction partner by refusing the offer. In the latter case, both players received 0 MU. We applied the strategy method again: the target participants decided whether to reject the unfair offer before knowing if that was the offer the first player chose.

In the *sharing game*, the target participant could either receive an amount for himself (leaving nothing for the interaction partner) or share the sum. There was no opportunity for the interaction partner to influence the outcome.

In the *nonsocial risk game*, the target participant played alone. In each of the eight rounds, he could choose between a low-risk gamble or a highly risky one. Next, the participant rolled a die to determine the outcome of the chosen gamble: Rolling a 1, 2, or 3 resulted in the higher outcome, whereas rolling a 4, 5, or 6 resulted in the lower outcome. Each participant played each variant once. The games were played in two sets. Each set involved a total of 12 decision rounds—6 were prosocial (2 rounds of the trust game, 2 of the trustworthiness game, and 2 of the sharing game), 2 were antisocial (punishment game), and 4 were nonsocial (nonsocial risk game)—and each round had a different payoff. To ensure that all decisions were made under acute psychosocial stress or under the effects of a control condition, we had target participants complete the first set of decisions immediately after the first stressor (or control condition) and the second set of decisions immediately after the second stressor (or control condition) (see Procedure). The set order was randomized. One example of each paradigm is shown in Fig 1.

The number of decisions reflecting trust, trustworthiness, sharing, or punishment was counted. Thus, for these measures, the maximum score was 4, and the minimum score 0. For the nonsocial risk game, 1 point was given for each decision favoring the risky gamble, which resulted in a minimum of 0 and maximum of 8. Monetary units earned from all decisions were disbursed after the experiment according to the following exchange ratio: 100 MU = 0.95€. The experiment was programmed and conducted with z-Tree software [58]. The Online Recruitment System for Economic Experiments (ORSEE) was used for recruiting and scheduling the group experiment sessions the [59].

### Psychological stress response

Psychological stress was measured with visual analogue scales [27]. Participants rated their level of stress, tension, physical symptoms, unpleasantness and pain at baseline (0 referring to the stressor’s onset), after the first and second parts of the decision paradigm, and +50 min after the second decision paradigm, respectively. We calculated one maximum increase value per subject from baseline to enable one dependent increase measure for each of the subjective ratings.

### Endocrine and autonomic stress response

We measured the cortisol stress response using a commercially available sampling device (salivette; Sarstedt, Nümbrecht-Rommelsdorf, Germany) eight times over the course of the experiment: at baseline (0 referring to the stressor’s onset), after the first part of the decision paradigm, after the second part of the decision paradigm, +10 min, +20 min, +35 min, +50 min, and +65 min relative to end of the decision paradigm. After each experimental session, samples were stored at -20°C. For biochemical analyses of free cortisol concentration, saliva samples were thawed and spun at 3000 rpm for 10 min to obtain 0.5–1.0 ml clear saliva with low viscosity. Salivary cortisol concentrations were determined using a commercially available chemiluminescence immunoassay (CLIA; IBL-International, Hamburg, Germany). Inter-and intraassay coefficients of variation were 8.4% and 4.6%, respectively. As described in the participants section, we defined responders in the both physical stress conditions (CPT and SECPT) according to Schwabe and colleagues [6]. We calculated the maximum increase in cortisol for each participant from baseline and defined responders by a cortisol increase of ≥ 2 nmol/l. This resulted in 17 participants in the CPT (out of 44 = 39% responders) and 21 participants in the SECPT (out of 45 = 47% responders). The WWT comprised 31 participants, the SEWWT group 32 (Table 1). Again, as we had done with the subjective stress response, we calculated the maximum increase per subject from baseline to enable one dependent increase measure for cortisol.

**Table 1:** Group characteristics with mean values ± SD in each group.

	*WWT*	*SEWWT*	*CPT*	*SECPT*	*p*
Age (years)	24.06 ± 5.01	24.75 ± 4.80	26.76 ± 5.13	23.67 ± 5.55	≥ 0.074
SIAS	19.58 ± 11.13	23.28 ± 10.25	22.18 ± 11.70	19.95 ± 6.89	≥ 0.162
BDI	6.19 ± 5.82	4.28 ± 4.03	5.06 ± 4.12	3.38 ± 3.37	≥ 0.059
IQ	105.16 ± 8.68	108.53 ± 10.82	106.65 ± 9.38	107.57 ± 8.07	≥ 0.314

*WWT = Warm Water Test*, *SEWWT = Socially Evaluated Warm Water Test*, *CPT = Cold Pressor Test*, *SECPT = Socially Evaluated Cold Pressor Test; SIAS = Social Interaction Anxiety Scale*, *BDI = Beck Depression Inventory*, *IQ = verbal IQ; p value resembles the lowest values for the each ANOVA model*.

Heart rate was measured as a marker of the sympathetic stress system using a wireless chest heart rate transmitter and wrist monitor recorder (Polar RS800 TM, Polar Electro, Finland). We recorded beat-to-beat heart rate data and calculated one-minute mean values: In order to control for potential group differences regardless of our stress manipulation we used five minute mean values and aggregated them within the instruction phase (of the interaction paradigm) of our experiment when participants were not yet aware in which stress condition they will be. For baseline heart rate we calculated the five minute mean values directly before the start of the first stress manipulation. For comparison of the baseline between our groups we aggregated these five values into one mean value. For the two stressor manipulations lasting three minutes each, we put five-minute mean values into the analyses in order to represent the course of the heart rate with a one-minute increase and recovery to the manipulation. This resulted in five values for the first part of the stressor and five for the second part thereof. For the two stress manipulations we additionally recorded the maximum increase in each participant by subtracting the mean heart rate baseline from the individual maximum within the four-minute window. Due to technical problems, heart rate data were obtainable from 91 participants in the final sample only (WWT: n = 28, SEWWT: n = 29, CPT: n = 16, SECPT: n = 18).

### Procedure

Participants were told to abstain from alcohol, caffeine, smoking, and medication intake 24h prior to the experiment. They should have eaten a standard lunch on the day of the experiment, and not have eaten after 4:00 pm. All participants received an email-reminder including these criteria the day before the experiment. They were randomly assigned to one of the four experimental conditions and invited in groups of 4 to 6. The two-hour sessions took place between 5:00 and 7:00 pm in order to control for diurnal variations in cortisol secretion.

**Fig 2:**
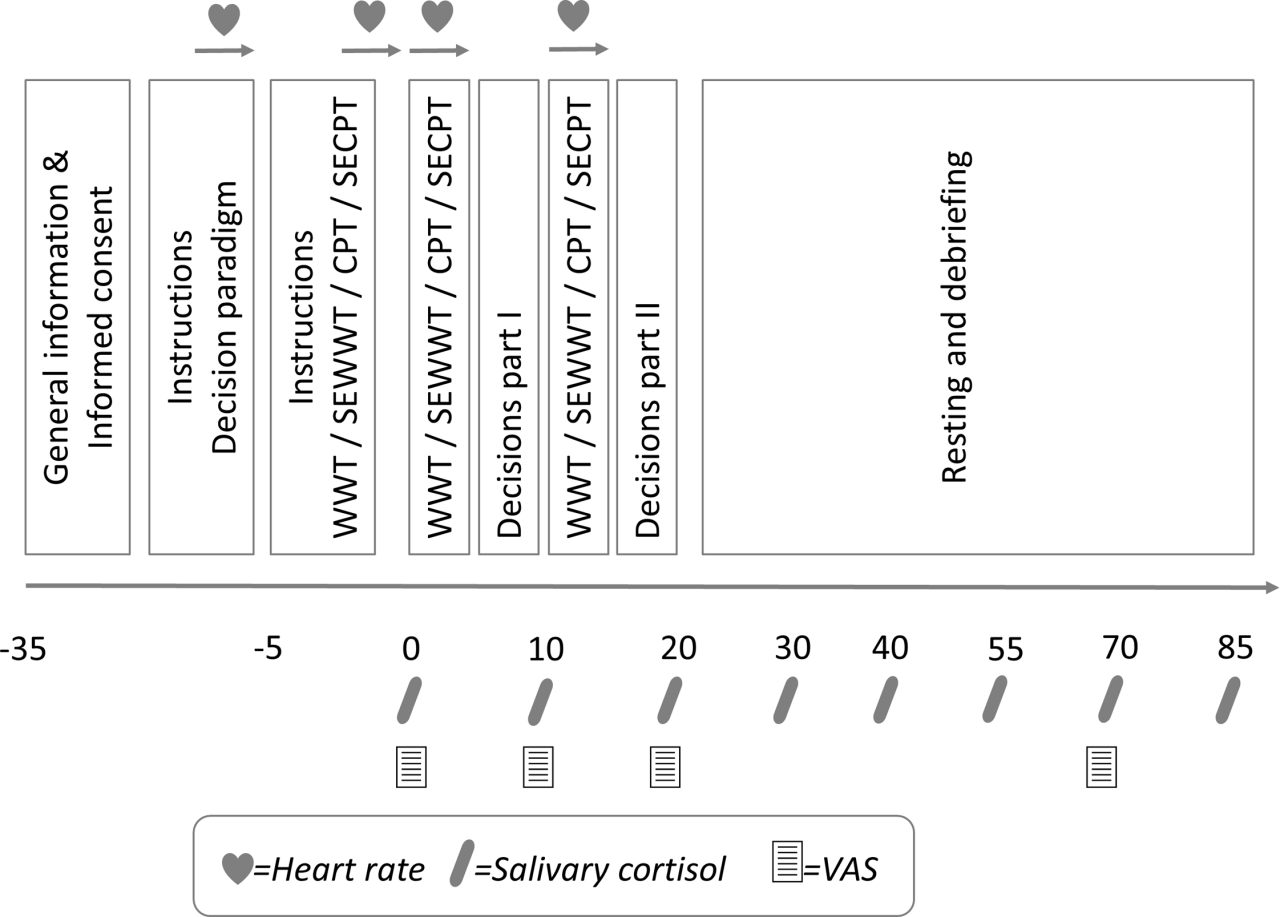
Timeline of the experiment.

Upon arrival at the laboratory, *target participants* were randomly assigned a number between 1 and 6 and seated individually accordingly to the number on their computer. They were not allowed to communicate. They read and signed the informed-consent forms, were introduced to the saliva sampling method, and each was provided with a heart rate device (Polar RS800TM, Polar Electro, Oy, Kempele, Finland). The participants then had to read instructions of the social and nonsocial interaction paradigms and were asked to complete control tasks (examples of each type of game). All participants responded to the control tasks correctly, indicating full understanding of the interaction procedure. They were then provided with instructions for one of the four different conditions: WWT, SEWWT, CPT or SECPT. After 5 min, they were guided to the test room and given a summary of the procedure. On their way to the test room, they came across the group of interaction partners waiting in front of the computer laboratory. In the test room, the sequence of activities was the following: 3 min of either warm water or cold water immersion with or without social evaluation (WWT, SEWWT, CPT or SECPT; stressor part I), first set of 12 decisions (5 min), 3 min of either warm water or cold water immersion with or without social evaluation (WWT, SEWWT, CPT or SECPT; stressor part II), and finally the second set of 12 decisions (5 min). The games were pencil-and-paper tasks. While participants completed their subjective ratings and gave their saliva sample, their decisions were entered at the specific computer in the computer laboratory. The interaction partners were already sitting there and had been instructed about the paradigms and made their decisions. After the target participants finished the procedure in the test room, they were guided to the computer laboratory and re-seated in their cubicles. Their previous decisions were matched to the interaction partners’ decisions by computer to determine everyone’s outcomes. The instructions about the decision paradigms guaranteed that all interactions would involve real human partners who would enter the laboratory after the stress manipulation. There was no deception involved. The interaction partners were invited to interact with the target participant to ensure real human interaction. All participants were provided with detailed written information and signed an informed consent form. All participants were reimbursed for their participation. This guaranteed the entire procedure’s complete credibility. After the target participants re-entered the computer lab, the results of each of their 24 decisions were presented on their computer screen, including the sum of their profits. The interaction partners then received the money they had earned (the converted sum of the outcomes for all 24 decisions plus the flat fee, which was paid out anonymously), and left the laboratory. Target participants had to stay in the lab until the last saliva sample was taken (+65 min after the end of the decision paradigm) and were then debriefed. Finally, they were paid the converted sum of the outcomes for all 24 decisions plus the flat fee. The study was approved by the ethics committee of the University of Freiburg, Germany. Written informed consent was obtained from all participants. The experiment’s timeline is found in Fig 2.

### Statistical analyses

Descriptive data (depression, social anxiety, age, and verbal IQ were compared using two-way analyses of variance (ANOVAs) with physical stress (cold water, warm water) and social stress (social evaluation, no social evaluation) as between-group factors. In order to control for the level of social anxiety, the SIAS score was entered as covariate in all analyses of covariance (ANCOVA) models: baseline cortisol, heart rate during instructions, heart rate baseline, and subjective-stress ratings were compared in three-way ANCOVAs with the factors physical stress and social stress. Cortisol and heart rate responses were analyzed using three-way ANCOVAs with repeated measures. The factors in these analyses were again physical stress, social stress, and time (repeated factor; 8 cortisol samples, 10 heart rate measurements). For the individual increases in heart rate we used a MANCOVA model and reported Wilks lamda Λ with the above reported factors and with the individual increase to the first and the second part of the stressor as two dependent variables. For the subjective stress responses, we calculated two-way ANCOVAs with the factors physical stress, social stress, and the maximum increase as dependent measure. The cortisol increase was also entered in a two-way ANCOVA with the factors physical stress and social stress. For the behavioral paradigms, again ANCOVAS were performed. In cases of heterogeneity of covariance (Mauchly test of sphericity), we determined the significance of the results of the repeated measures ANOVAs and ANCOVAs following Greenhouse-Geisser corrections. Effect sizes are reported as η_p_^2^ for ANOVAs and ANCOVAs. Post-hoc independent samples t-tests were run to detect specific differences between conditions. To explore the potential underlying mechanisms of behavioral effects of our stress manipulations, we decided to conduct stepwise regression models within each group with the behavioral variables as criteria and the subjective as well as biological stress measures as predictors. For the heart rate increase we calculated one mean measure by combining the two max increase mean values. Data were analyzed using SPSS Version 21 and 24. All tests were two-sided, with the level of significance set at *p* < .05.

## Results

### Psychological trait and baseline measures

The four groups did not differ significantly in their level of social anxiety, depressive symptoms, age, or verbal IQ (all *p* > 0.050) (Table 1). With regard to age and depressive symptoms (BDI) we observed differences on a trend level. Since social anxiety is correlated to depressive symptoms [60], we decided to add the BDI as another covariate into all of the following statistical models in order to control for possible confounding effects. The results reported below will therefore include social anxiety and depressive symptoms as covariates.

In addition, the groups did not differ in baseline levels of cortisol, heart heart rate during instructions, subjective stress, unpleasantness, physical symptoms, tension, or pain, respectively (all p ≥ 0.100). There was a trend towards an effect of social stress for baseline heart rate (F(1,85) = 3.17, p = 0.079, ηp2 = 0.036). For an overview of the baseline levels, please see Table 2.

**Table 2:** Baseline characteristics with mean values ± SD in each group.

	WWT	SEWWT		CPT		SECPT		p
Cortisol	3.73 ± 2.75	4.40 ± 4.36		2.95 ± 1.87		3.95 ± 4.85		≥ 0.292
Heart Rate during instructions	83.03 ± 14.61	83.23 ± 10.15		81.75 ± 5.43		84.75 ± 14.98		≥ 0.548
Heart Rate baseline	70.10 ± 10.06	73.71 ± 9.87		69.34 ± 5.69		74.21 ± 13.82		≥ 0.079
VAS Stress	10.13 ± 12.67	11.28 ± 12.31		8.88 ± 11.92		8.33 ± 8.87		≥ 0.390
VAS Unpleasantness	7.39 ± 9.98	8.63 ± 8.56		7.71 ± 8.58		6.62 ± 6.78		≥ 0.489
VASPhysical symptoms	11.35 ± 13.89	9.94 ± 11.40		9.35 ± 10.39		6.48 ± 7.85		≥ 0.254
VAS Tension	11.61 ± 14.10	11.81 ± 12.98		11.00 ± 11.49		10.24 ± 9.43		≥ 0.632
VAS Pain	6.06 ± 14.631	1.75 ± 5.304		1.59 ± 2.575		4.81 ± 9.293		≥ 0.100

*WWT = Warm Water Test*, *SEWWT = Socially Evaluated Warm Water Test*, *CPT = Cold Pressor Test*, *SECPT = Socially Evaluated Cold Pressor Test; VAS = Visual Analog Scale; p value resembles the lowest values for the each ANCOVA model*.

### Psychological stress responses

**Fig 3:**
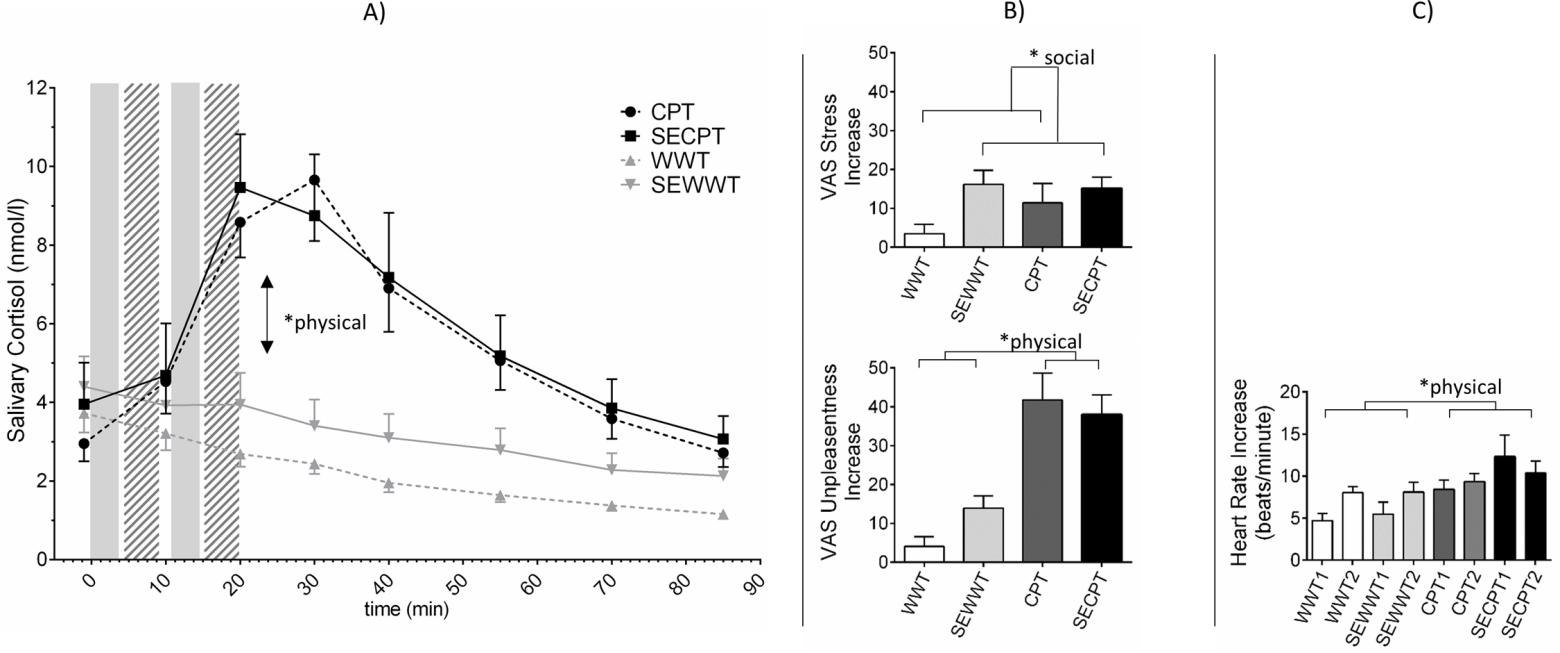
**A) mean values of salivary cortisol; solid bars: time of water immersion; shaded bars: decision making; B) mean values of increases in subjective stress and unpleasantness measured with VAS; C) increases in heart rate to the first and the second stressor.** Error bars indicate standard errors of the mean; WWT = Warm Water Test, SEWWT = Socially Evaluated Warm Water Test, CPT = Cold Pressor Test, SECPT = Socially Evaluated Cold Pressor Test. * indicate significant differences with p≤0.05.

We detected a significant effect of social stress on the subjective stress increase (F(1,95) = 5.55, p = 0.021, ηp2 = 0.055) (Fig 3B) and increase in tension (F(1,95) = 7.901, p = 0.006, ηp2 = 0.077) with higher levels in the social stress condition. Physical stress led to stronger increases in physical symptoms (F(1,95) = 28.05, p<0.001, ηp2 = 0.228), unpleasantness (F(1,95) = 58.41, p<0.001, ηp2 = 0.381) (Fig 3B), and pain (F(1,95) = 28.87, p<0.001, ηp2 = 0.233). The covariate social anxiety modulated the increase in subjective stress (F(1,95) = 3.40, p = 0.068, ηp2 = 0.035), and tension (F(1,95) = 3.60, p = 0.061, ηp2 = 0.036) on a trend level but not the increase in physical symptoms (p = 0.433), unpleasantness (p = 0.113) or pain (p = 0.443). The higher the level of social anxiety, the higher the subjective response in terms of stress and tension. There was no interaction between physical and psychological stress (all p>0.1). For depressive symptoms there were higher increases with higher levels of depressive symptoms on a trend level (F(1,95) = 3.88, p = 0.052, ηp2 = 0.039) (Fig 3B shows the increase in subjective stress and unpleasantness. The course of all subjective responses is presented in S2 Fig. All stastistical values of all variables can be found in S2 Table.

### Physiological stress responses

All groups presented similar baseline cortisol levels and heart rates (all p<0.001) (Table 2). We noted a significant increase in salivary cortisol over time (F(2.77, 262.95) = 9.52, p<0.001, ηp2 = 0.091) as well as a time x physical stress interaction (F(2.77, 262.95) = 36.91, p<0.001, ηp2 = 0.280), showing higher increases in the two physical stress conditions. There was also a main effect of physical stress (F(1, 95) = 17.193, p<0.001, ηp2 = 0.153) (Fig 3A). Social anxiety and depressive symptoms did not modulate the cortisol stress response. There was no time x physical x social stress interaction, nor any main effect from the physical x social stress interaction. The increase in cortisol was significantly higher in the physical stress conditions (F(1,95) = 83.19, p<0.001, ηp2 = 0.467). Again, neither social anxiety nor depression did modulate the increase in cortisol. We observed no effect from social stress or physical x social stress interaction.

Regarding the heart rate response to the various stressors: neither social anxiety nor depression did not modulate the response significantly. There was a significant increase in heart rate over time (F(4.96, 421.50) = 3.48, p = 0.004, ηp2 = 0.039) and a significant time x physical stress effect (F(4.96, 421.50) = 8.95, p<0.001, ηp2 = 0.095) with higher heart-rate increases in the physical stress condition. In addition, social stress revealed significant influence over time (time x social stress: F(4.96, 421.50) = 2.85, p = 0.015, ηp2 = 0.032) with higher increases over time in the social stress conditions. Moreover, there was a significant three-way interaction of time, social stress, and physical stress (F(4.96, 421.50) = 2.72, p = 0.020, ηp2 = 0.031) with the highest increases in heart rate in the SECPT condition. We also noted a trend towards a main social-stress effect (F(1,85) = 3.89, p = 0.052, ηp2 = 0.044), with overall higher heart-rate levels in the social stress conditions. There was no main effect of physical stress on heart rate. The MANCOVA model with the individual increases in heart rate yielded the following results: there was again no effect of social anxiety or depression. Physical stress led to significantly higher increases in heart rate (F(2, 84) = 6.00, p = 0.004, Wilk's Λ = 0.875 ηp2 = 0.125). With respect to the maximum increase we did not find a significant effect of social stress or a significant interaction between physical and social stress (Fig 3C).

### Effects of social and physical stress on prosocial behavior, punishment, and nonsocial risk

**Fig 4:**
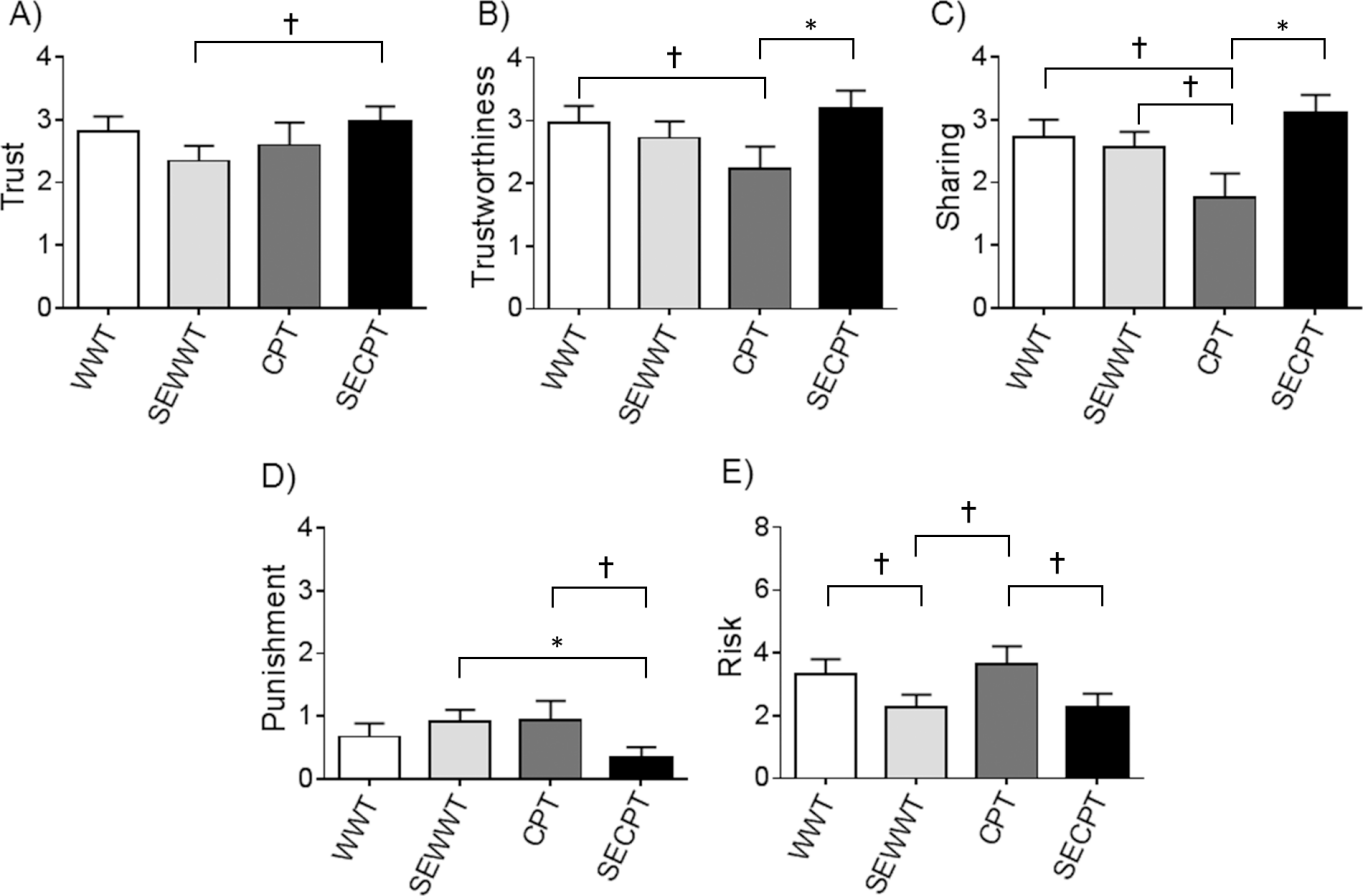
**Mean score as a function of condition for A) trust B) trustworthiness C) sharing D) punishment and E) nonsocial risk**. Error bars indicate standard errors of the mean; * indicate post-hoc t tests with a p≤0.05, † with a p ≤0.10; WWT = Warm Water Test, SEWWT = Socially Evaluated Warm Water Test, CPT = Cold Pressor Test, SECPT = Socially Evaluated Cold Pressor Test.

Regarding the prosocial behaviors trust, trustworthiness, and sharing, we identified significant modulation by the covariate social anxiety only for trust, reflecting higher levels of trust with higher levels of social anxiety (F(1,95) = 12.52, p = 0.001, ηp2 = 0.116). Depression had no significant influence. We noted a trend towards higher levels of sharing in the social stress condition (F(1,95) = 3.79, p = 0.055, ηp2 = 0.038). While social stress or physical stress alone reduced prosocial behavior on the descriptive level, combining the two factors restored the level of prosociality: this means that one stressor alone (either social evaluation or cold water) reduced prosociality, while a combination of the two stressors triggers a level of prosociality similar to that in the group with no stressor. This result is reflected by a social x physical stress interaction with significant effects consistent for all three prosocial behaviors: trust (F(1, 95) = 4.49, p = 0.037, ηp2 = 0.045), trustworthiness (F(1,95) = 5.01, p = 0.027, ηp2 = 0.050) and sharing (F(1, 95) = 5.94, p = 0.017, ηp2 = 0.059). Regarding punishment: neither social anxiety nor depression did not modulate punishment behavior, but there was a trend towards the interaction between physical and social stress (physical x social stress: F(1,95) = 3.56, p = 0.062, ηp2 = 0.036). While social stress or physical stress alone increased punishment, the combination of the two factors again reduced the level of punishment. Regarding nonsocial risk behavior, again social anxiety or depression did not significantly modulate risky choices, but social stress did exhibit a significant effect: participants displayed lower levels of nonsocial risk if they were socially evaluated compared to the non-social condition (social stress: F(1,95) = 4.97, p = 0.028, ηp2 = 0.050). Physical stress revealed no significant effect on nonsocial risk behavior. Post-hoc t-tests tended to indicate difference towards lower trust in the SEWWT compared to the SECPT condition (t(51) = 1.707, p = 0.094). For trustworthiness we observed a trend towards lower levels in the CPT compared to the WWT condition (t(46) = 1.680, p = 0.100) and significantly higher levels in the SECPT compared to the CPT condition (t(36) = 2.126, p = 0.040). For sharing there were lower levels in the CPT condition than the WWT condition (t(46) = 1.976, p = 0.054) as well as compared to the SEWWT condition (t(47) = 1.851, p = 0.070), both on a trend level. The CPT condition revealed significantly lower levels of sharing than the SECPT condition (t(36) = 2.828, p = 0.008). For punishment there were significantly lower levels in the SECPT than the SEWWT condition (t(51) = 2.178, p = 0.034) and a trend towards lower levels of punishment in the SECPT than the CPT condition (t(36) = 1.741, p = 0.093). Risk was lower on a trend level in the SEWWT compared to the WWT condition (t(61) = 1.713, p = 0.092), lower in the SEWWT than the CPT condition (t(47) = 1.995, p = 0.052) and lower in the SECPT than the CPT condition (t(36) = 1.955, p = 0.058). All behavioral paradigm results are shown in Fig 4.

For exploratory analyses of the potentially underlying mechanisms of the different stress systems and their magnitude, we calculated stepwise regression models in each of the four groups for each of the five behavioral variables. We entered all five VAS Increase Variables, the maximum cortisol increase and the mean heart rate increase into our models.

VAS Unpleasantness was positively related to trust in the SEWWT and CPT condition. In the SECPT condition two significant models were depicted. The first model including a negative relationship between cortisol increase and trust and in the second model a negative relationship between increase in cortisol as well as the VAS Stress increase and trust which revealed the highest adjusted R^2^ (S7 Table). The cortisol increase was also negatively related to trustworthiness in the SECPT condition. No other model appeared to show significance for trustworthiness (S8 Table). For sharing, we again found a negative relation for the cortisol increase in the SECPT group. In addition the increase in heart rate was negatively related to sharing in the CPT condition (S9 Table). For punishment only one model was validated which shows a negative relationship between the increase in heart rate and the amount of punishment in the WWT group (S10 Table). With respect to risk behavior there was no significant model at all. Taken together these results do not reveal clear consistent patterns.

## Discussion

This is the first study to investigate the effects of social stress, physical stress, and social anxiety on social behavior. Social stress increased subjective stress and tension, whereas physical stress increased physical symptoms, unpleasantness, and pain. There was no interaction between social and physical stress with regard to the subjective stress ratings. Participants high in social anxiety reported stronger increases in stress and tension on a trend level, but did not differ in their reported increases in unpleasantness, physical symptoms or pain. Cortisol was increased by physical stress, but there was no stronger effect for the interaction of social and physical stress and no effect of social stress alone. In terms of the cardiovascular response there were increases by physical stress and social stress over time, but again no interaction. Social anxiety revealed no association with the physical stress response. The level of depressive symptoms did not influence the psychobiological stress response significantly.

For our baseline measure before the start of the stress manipulation we documented slightly higher levels for heart rate in the social stress conditions on a trend level. This may be interpreted as an anticipatory stress response in this system as the introduction to the stress manipulation has already been given at that point.

For the behavioral variables our study demonstrates that the physical and social components of stress exposure interacted to modulate social behavior in men. In particular, social stress alone reduced prosocial behavior, while the lowest levels of prosocial behavior became apparent following physical stress exposure alone. Importantly, combining both stressors led to the restoration of trust, trustworthiness, and sharing, as well as a trend towards less punishment compared to the physical stress condition. We cannot interpret the effects of the social stress or physical stress separately, since their effects did not appear significant in our model. But the significant interactions are evidence that the behavioral effects of acute stress are stressor modality-dependent. The effects of social evaluation on behavior differ depending whether a person is subjected to a cold stressor at the same time or not (and vice versa).

In addition we found that social stress alone reduced risky nonsocial decisions and increased sharing behavior. Moreover, social anxiety modulated trust behavior significantly, with higher social anxiety levels being associated with increased trust. Depressive symptoms had no significant effect on any behavioral variable.

Group-to-group comparisons confirmed the aforementioned results: among all four groups the clearest finding was lower levels of trustworthiness and sharing in the CPT versus the SECPT group. On a trend level the group-to-group comparisons confirm the finding that social and physical stress alone slightly reduce prosocial behavior, while their combination restores prosociality. These results should be interpreted with caution because of possible α error cumulation due to multiple comparisons and the fact that the level of social anxiety was not taken into account.

Our findings highlight the notion that men seek positive social encounters when faced with threatening circumstances [27,61], which supports the tend-and-befriend hypothesis in the context of stress-induced social behavior [29]. But this is only the case when individuals face a stressor with a specific threat pattern: in our study it was the combination of a physical (cold water) and social component (social evaluation). When confronted with a physical (non-social) stressor, participants exhibited reduced levels of prosocial behavior, thus replicating a recent study’s findings [62].

Social evaluation is a potent stressor known to lead to stronger cortisol increases when combined with other stressor elements [5]. It was added to the cold pressor stressor to increase its impact on the stress response and thus form a new method for stress induction, the SECPT [6,56]. We expected it to demonstrate efficacy in humans depending on the level of social anxiety, since social evaluation is the core annoyance these individuals try to avoid and fear [63]. Although we found effects on the subjective stress response (higher increases in subjective stress and tension), we noted only a trend towards an increase in sharing behavior and no other social-evaluation effects on socially interactive behaviors alone. One could speculate that our social evaluation manipulation was not strong enough compared to the variant used in the TSST-G [49]. This could be responsible for the lack of effect on the cortisol stress response. Regarding the combination with physical stress, we noted effects that might be comparable to effects observed with other social stressors [27]. We could not prove this effect to be driven by the cortisol increase as previous studies demonstrated [23,36,64], but this might be due to the stressors varying in intensity, or to qualitative differences in the psychophysiological stress responses to diverse stressor paradigms–all factors deserving investigation in future studies. Interestingly, we detected significantly fewer nonsocial high-risk choices under social stress or social evaluation, a result that falls in line with studies showing that even pictures of human eyes may already induce prosocial and normative behavior [65–67].

Social anxiety modulated several aspects of the subjective stress response but not the physical stress response, findings that concur with studies reporting discordance between physiological and subjective stress parameters [42,45] in social-anxiety patients. Our results underline the importance of studying the psychobiological mechanisms that trigger the effects of stress on behavior, as subjective differences in situational interpretations may be important even when they are not accompanied by biological differences, e.g. in cortisol. Moreover, social anxiety modulated trust in our participants, with the highest levels of social anxiety being associated with the highest trust scores, a finding in line with a study on patients with social anxiety disorder that reported more submissive behavior in socially anxious individuals [68]. Participants high in social anxiety may trust more because they have too little self-confidence to withhold their trust. They may not differ in their levels of sharing or trustworthiness since such decisions are one-shot decisions that do not depend on another participant’s decision. The sharing game resembles a dictator game, whereas our trust game may be better compared to an ultimatum game of slightly different structure. There is evidence that behavior in these games differs with higher prosociality in the ultimatum game compared to the dictator game where no other decision or response than the proposer´s is accountable [69,70]. One explanation for this difference beyond differences in fairness motivation may be the fear of rejection that comes into play in the ultimatum and trust game, but not in the dictator game. Participants with high levels of social anxiety may have trusted more because they harbor an elevated fear of rejection and not because they are necessarily more motivated to demonstrate greater general fairness. Future studies should investigate further the role of maladaptive beliefs that could lead to differences in prosocial behavior in social anxiety [71]. Furthermore, comparison between patients with social anxiety disorder and subclinical levels of social anxiety would lead to deeper understanding of the modulation of social behavior under stress on health and pathology terms.

With regard to the possible underlying mechanisms of our behavioral results: we detected no clear pattern in our results. We found positive associations for the increase in VAS Unpleasantness with the increase in the SEWWT and the CPT condition what would go in line with the concept of tend-and-befriend response. Surprisingly, we found negative associations for the increase in cortisol with trust, trustworthiness and sharing in the SECPT condition. Also VAS Stress increase was related negatively to trust in the SECPT condition. Maybe this reflects possible magnitude effect. As all participants within the SECPT condition show stress increases, it may be that prosocial behavior is shown especially by the moderate to low responders while high responders may tend to reduce their prosocial behavior. This was accompanied by a negative relation between heart rate increase and sharing in the CPT condition. For the WWT condition there was a negative association between heart rate increase and punishment. Since these analyses only have exploratory character and bear methodological limitations (e.g. small sample size) we will not draw conclusions here. Because we had some data loss in the heart rate condition as described in the methods section, the regression analyses included only these participants with respect to all other stress measures due to listwise exclusion. We recommend interpreting these findings with caution; it will be up to future studies to reveal the underlying mechanisms in study designs enabling a causal inference, e.g. by activating or blocking different physiological stress systems with pharmaceutical agents [64]. In addition sample sizes will need to be increased substantially.

The present study shows that the behavioral effects of stress on social behavior depend on the stressor’s quality that can more intensely activate either the fight-or-flight or tend-and-befriend response pattern. Our results could lead one to speculate that both concepts characterize distinct variants of stress-related behavioral action: whether one or both behavioral aspects are activated depends on the quality of the stressor, specific behavioral options, and on individual characteristics (eg, the trait level of social anxiety). In addition, a direct comparison of men and women in one study would be needed to clarify any gender-associated dominance of one or the other behavioral concept. In the current study, we did identified no significantly increased prosocial behavior in the stress (SEWWT, CPT, SECPT) compared to the control condition (WWT) [27]. One might speculate that this is due to the quality of the stress induction and responses in our study. The SEWWT and SECPT differ from the TSST-G in its socio-evaluative component with lower levels of social threat in the SEWWT and SECPT than in the TSST-G, which requires the participant to present himself and perform on several levels. This involves deeper ego-involvement and the potential for personal embarrassment. On the other hand, the SECPT includes a physical stressor. As these situations and behaviors differ so strongly from one another, future studies will need to test a wider range of stressors of varying intensity and qualities on social behavior in order to identify any dose-dependent effects of stress on behavior, as well as which specific qualitative aspects modulate social behavior under stress. Interestingly, even traumatic events seem to have the potential to prompt prosocial behavioral tendencies in people [72], a factor that might initially appear strange: although traumatic events (reflecting strong psychobiological stressors) bear the risk of developing posttraumatic stress disorder, most affected people exhibit resilience [73,74]—another influencing factor linked to social support. Better understanding of the interplay between differential contextual and psychopathological factors of stress, their psychobiological underpinnings, and their behavioral consequences might help us to understand resilience better.

Physical and social stress interact to modulate social interaction in men, and social stress alone reduces nonsocial risk behavior. A recent meta-analysis of imaging data on physical and psychosocial stress documented that physical and psychosocial stressors lead to similar subjective and physiological results, but display different underlying brain activation patterns. While physical stress is associated with motoric fight-or-flight responses, psychosocial stress leads to a shift towards emotion regulation, goal-directed behavior, and a reduction in reward processing [75]. Our results support that notion with the reductions in prosocial behavior in the CPT condition, which could be interpreted as fight-or-flight behavior. Interestingly, the psychosocial stress component seems to compensate this effect and restore prosocial tendencies irrespective of the level of social anxiety. Imaging studies should reveal the underlying brain activity patterns in the SECPT compared to CPT and a psychosocial stress like the Montreal Imaging Stress Task (MIST) [76].

Our study shows that both the fight-or-flight and tend-and-befriend tendencies are part of the human behavioral repertoire under stress, and that they may be differentially activated. Deeper insights into the underlying mechanisms will inspire researchers and clinicians to adopt more specific diagnostic and treatment approaches for patients with social anxiety disorder to successfully tailor individual therapy approaches.

## Supporting information

S1 Fig All payoff structures for the games assessing prosocial behaviors and antisocial and nonsocial risk behaviors. The target participant and that participant’s interaction partner are represented by a red P and black IP, respectively (interaction partners were not in either the stress or control condition). The pairs of numeric values are examples of the outcomes (in monetary units) received by the target participant (red values) and interaction partner (black values). In the nonsocial risk game, target participants rolled a die, and its value determined which outcome resulted.
(PPTX)

S2 Fig Course of subjective increases for all VAS. Mean values and standard errors of the mean; solid bars: time of water immersion; shaded bars: decision making; A) stress B) unpleasantness C) tension D) physical symptoms E) pain; WWT = Warm Water Test, SEWWT = Socially Evaluated Warm Water Test, CPT = Cold Pressor Test, SECPT = Socially Evaluated Cold Pressor Test.
(PDF)

S1 Table Stastical values of baseline characteristics. F an p values of baseline characteristics.
(PDF)

S2 Table Stastical values of psychological stress response. F an p values of psychological stress response.
(PDF)

S3 Table Stastical values of physiological stress response with repeated measures–Within subject results. F and p values of physiological stress response within subjects results.
(PDF)

S4 Table Stastical values of physiological stress response with repeated measures–Between subjects main effects. F and p values of physiological stress response between subjects effects.
(PDF)

S5 Table Stastical values of physiological stress response–maximum increases of cortisol and heart rate. F values, Wilk'sΛ and p values.
(PDF)

S6 Table Stastical values of ANCOVA models for behavioral paradigms. All F and p values.
(PDF)

S7 Table Stepwise regression to explore relationships between of stress systems and trust. All parameters of significant models.
(PDF)

S8 Table Stepwise regression to explore relationships between of stress systems and trustworthiness. All parameters of significant models.
(PDF)

S9 Table Stepwise regression to explore relationships between of stress systems and sharing. All parameters of significant models.
(PDF)

S10 Table Stepwise regression to explore relationships between of stress systems and punishment. All parameters of significant models.
(PDF)

S11 Table Stepwise regression to explore relationships between of stress systems and risk. All parameters of significant models.
(PDF)
