# Supplementary material for: Acute social and physical stress interact to influence social behavior: The role of social anxiety
Source: PLoS One. 2018 Oct 25;13(10):e0204665. doi: 10.1371/journal.pone.0204665 (PMC6201881; doi:10.1371/journal.pone.0204665)
Supplement: S1 Fig — The target participant and that participant’s interaction partner are represented by a red P and black IP, respectively (interaction partners were not in either the stress or control condition). The pairs of numeric values are examples of the outcomes (in monetary units) received by the target participant (red values) and interaction partner (black values). In the nonsocial risk game, target participants rolled a die, and its value determined which outcome resulted. (PPTX) [file pone.0204665.s001.pptx]

## Slide 1
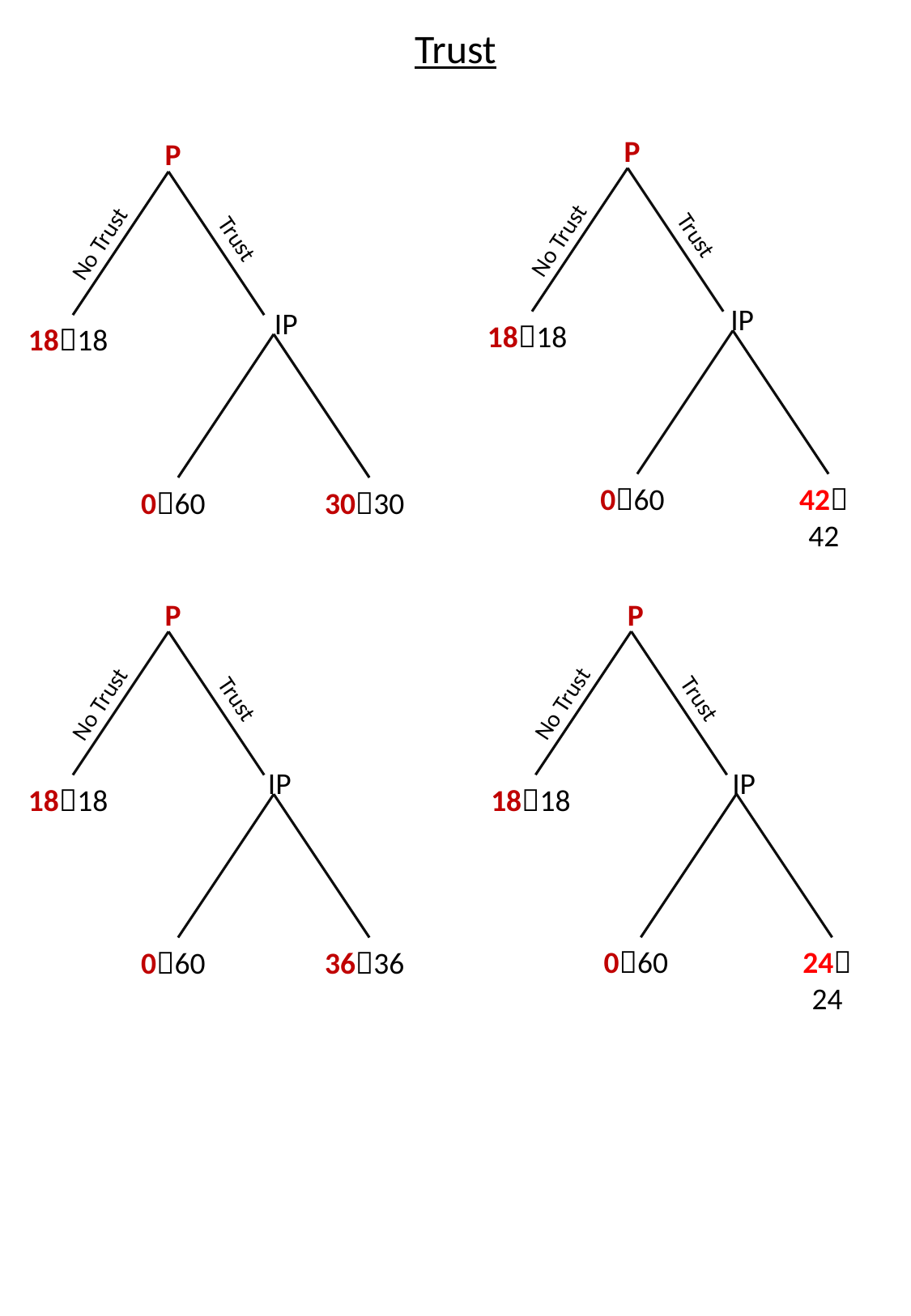

Trust
P
Trust
No Trust
IP
1818
060
42 42
P
Trust
No Trust
IP
1818
060
3030
P
Trust
No Trust
IP
1818
060
24 24
P
Trust
No Trust
IP
1818
060
3636

## Slide 2
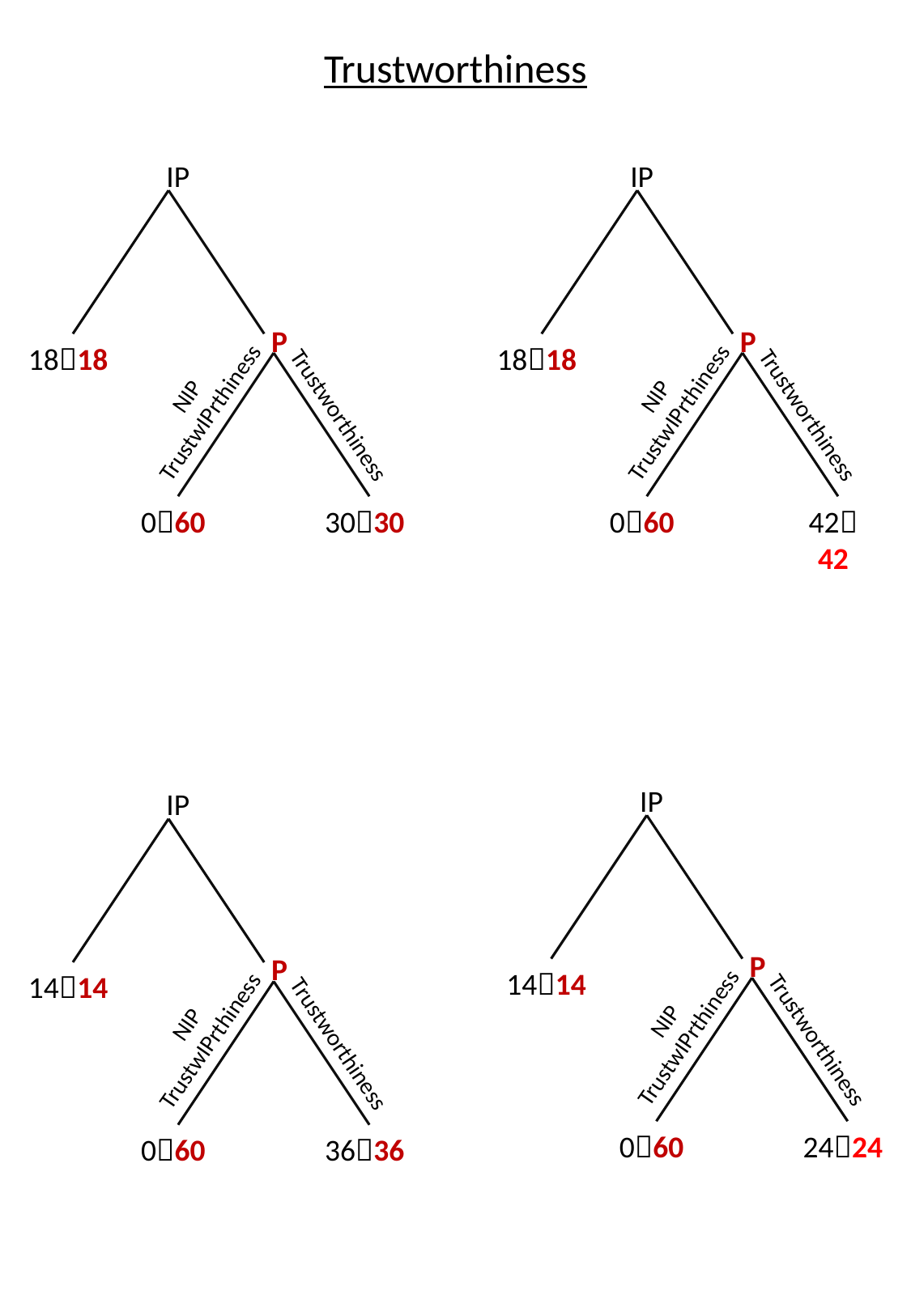

Trustworthiness
IP
P
1818
NIP TrustwIPrthiness
Trustworthiness
060
3030
IP
P
1818
NIP TrustwIPrthiness
Trustworthiness
060
42 42
IP
P
1414
NIP TrustwIPrthiness
Trustworthiness
060
2424
IP
P
1414
NIP TrustwIPrthiness
Trustworthiness
060
3636

## Slide 3
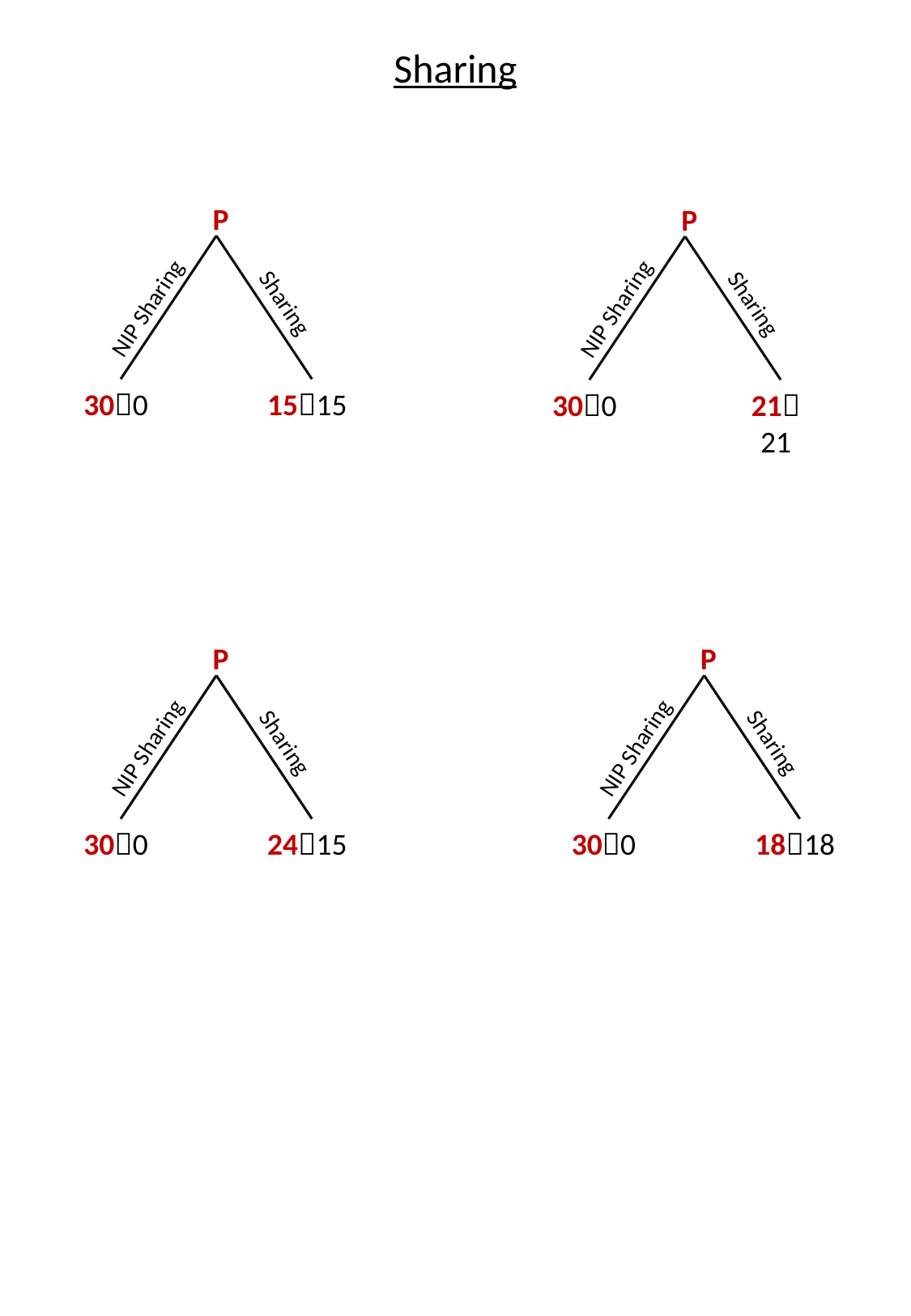

Sharing
P
Sharing
NIP Sharing
300
1515
P
Sharing
NIP Sharing
300
21 21
P
Sharing
NIP Sharing
300
2415
P
Sharing
NIP Sharing
300
1818

## Slide 4
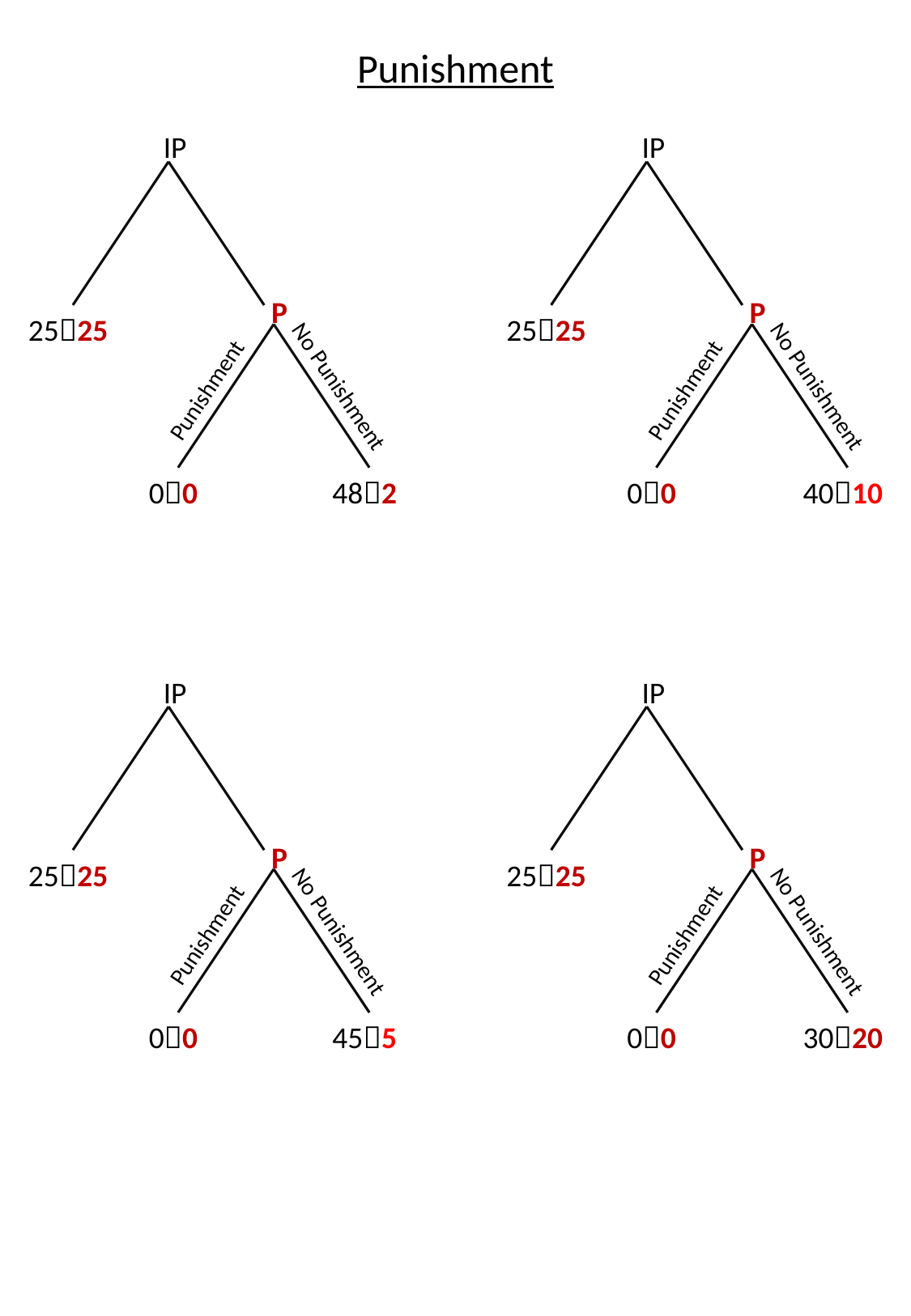

Punishment
IP
P
2525
No Punishment
Punishment
00
482
IP
P
2525
No Punishment
Punishment
00
4010
IP
P
2525
No Punishment
Punishment
00
455
IP
P
2525
No Punishment
Punishment
00
3020

## Slide 5
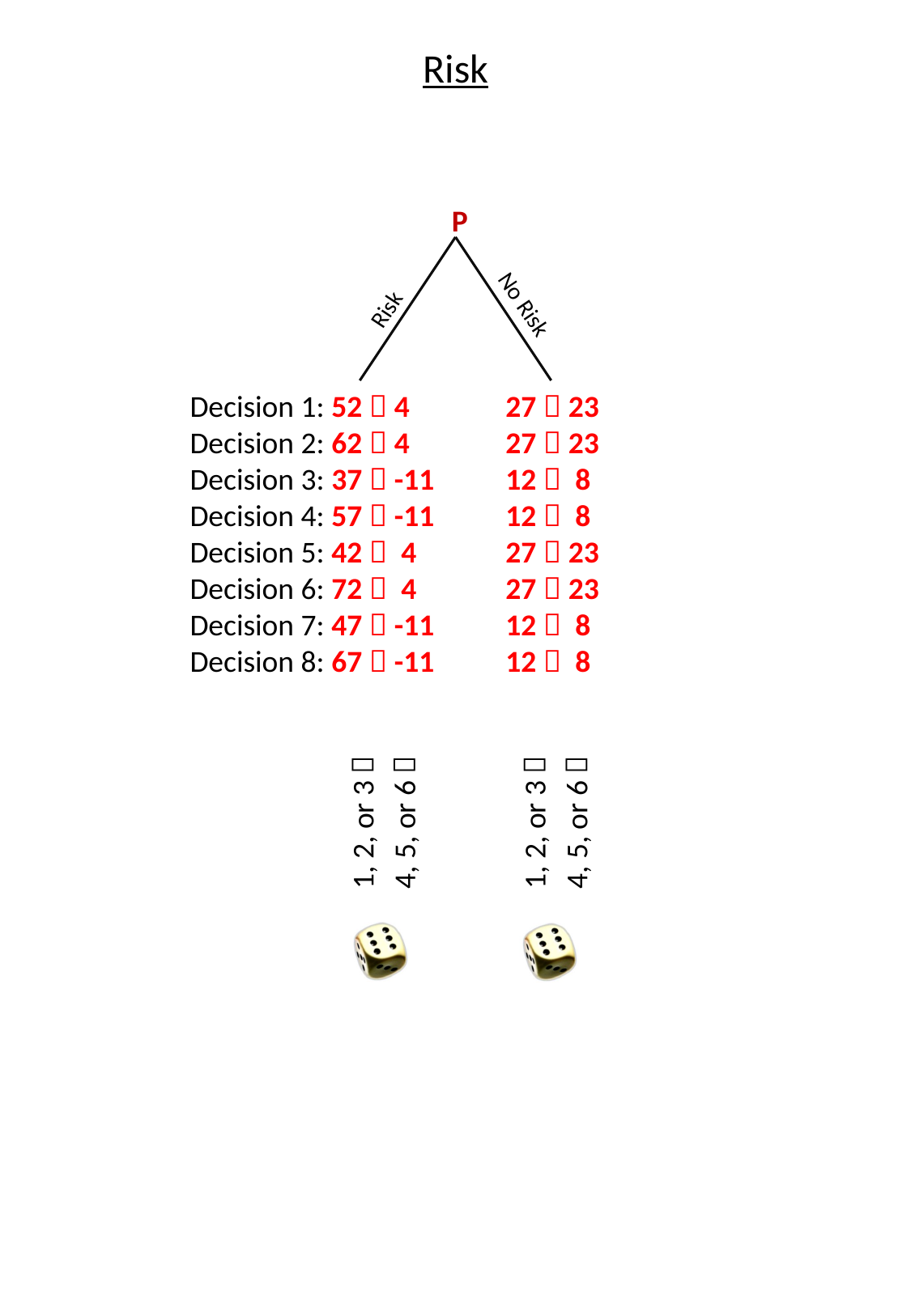

Risk
P
No Risk
Risk
Decision 1: 52  4
Decision 2: 62  4
Decision 3: 37  -11
Decision 4: 57  -11
Decision 5: 42  4
Decision 6: 72  4
Decision 7: 47  -11
Decision 8: 67  -11
27  23
27  23
12  8
12  8
27  23
27  23
12  8
12  8
 1, 2, or 3 
 1, 2, or 3 
 4, 5, or 6 
 4, 5, or 6 
