# Supplementary figures and images for: Acute social and physical stress interact to influence social behavior: The role of social anxiety
Source: PLoS One. 2018 Oct 25;13(10):e0204665. doi: 10.1371/journal.pone.0204665 (PMC6201881; doi:10.1371/journal.pone.0204665)

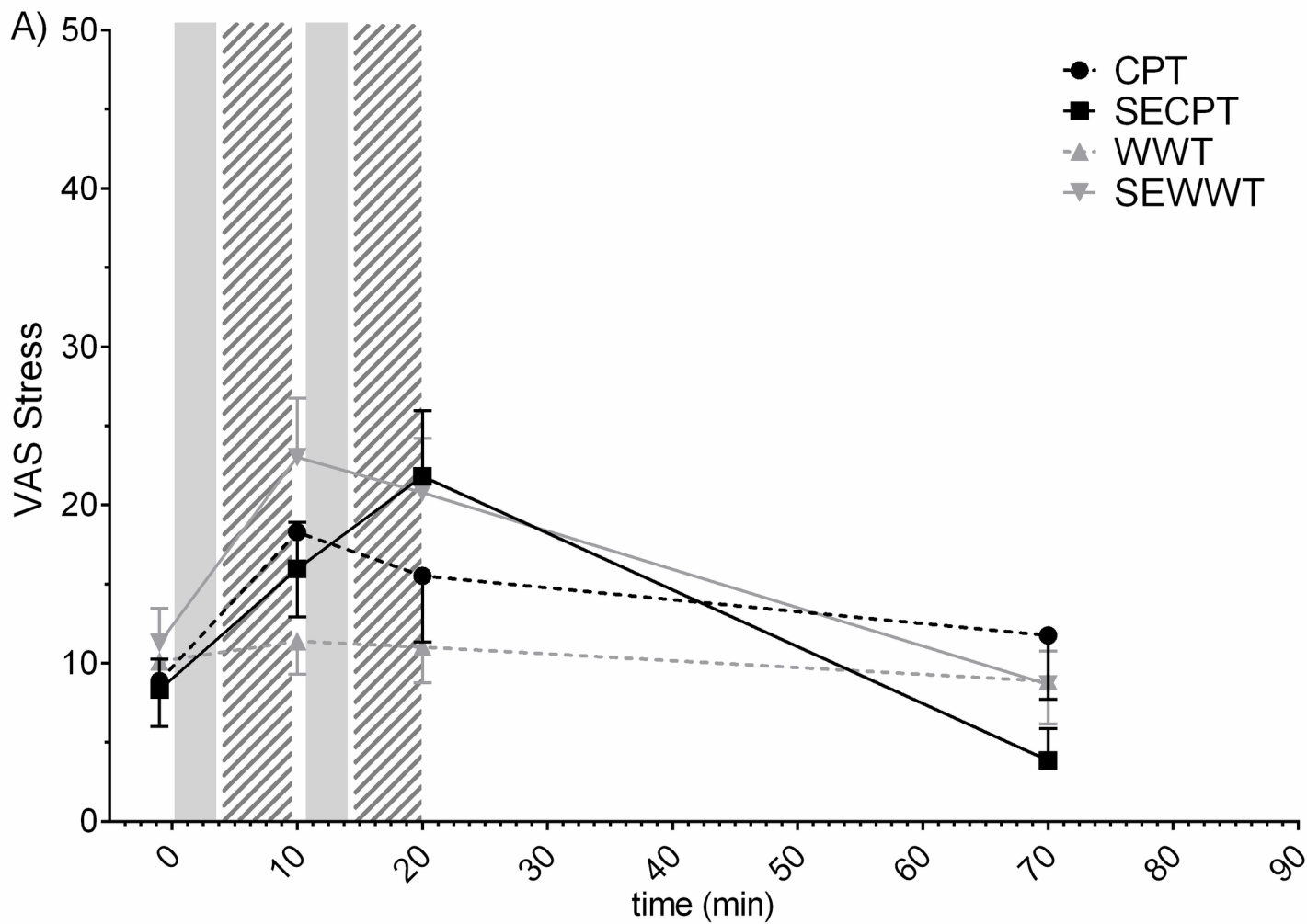

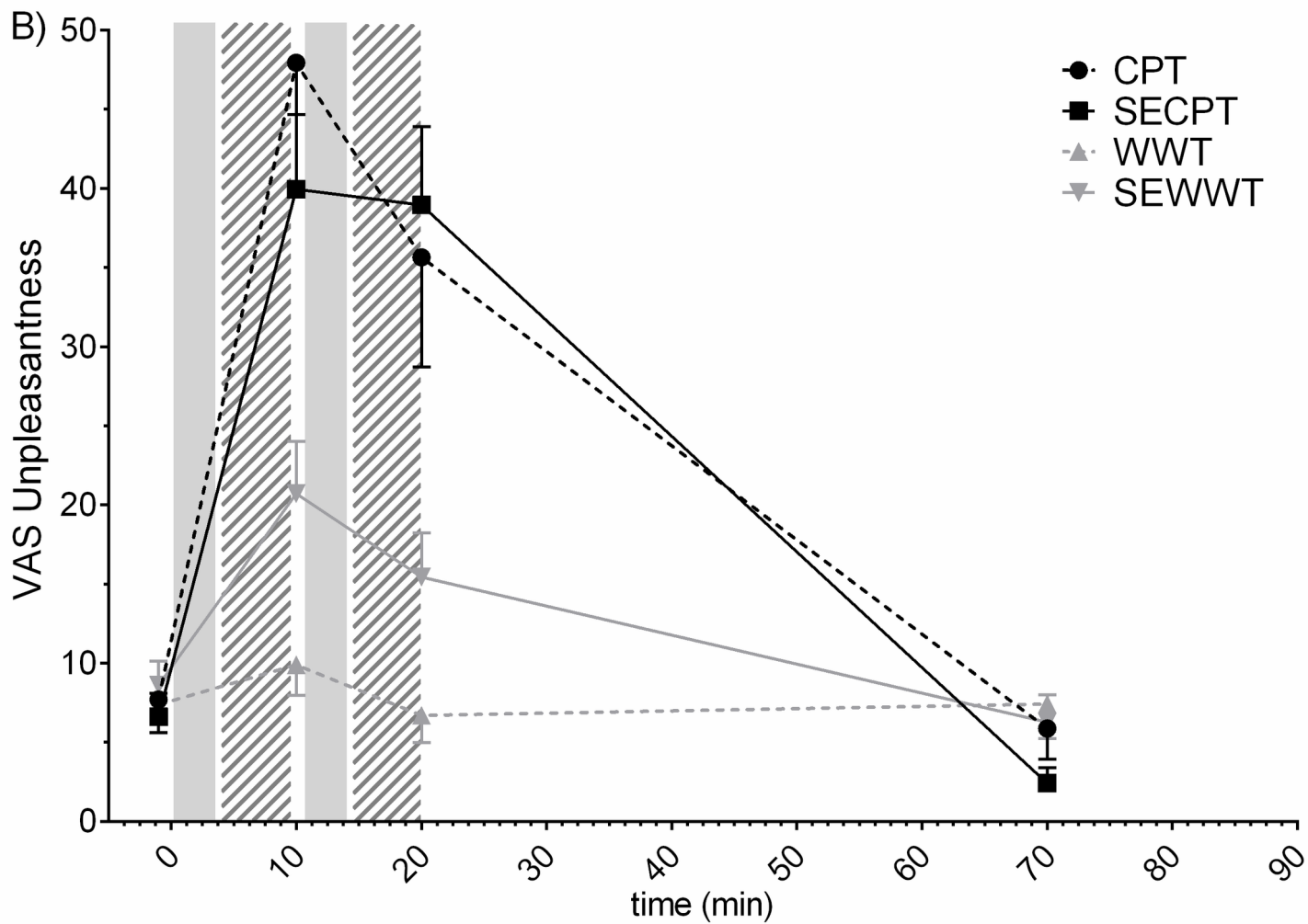

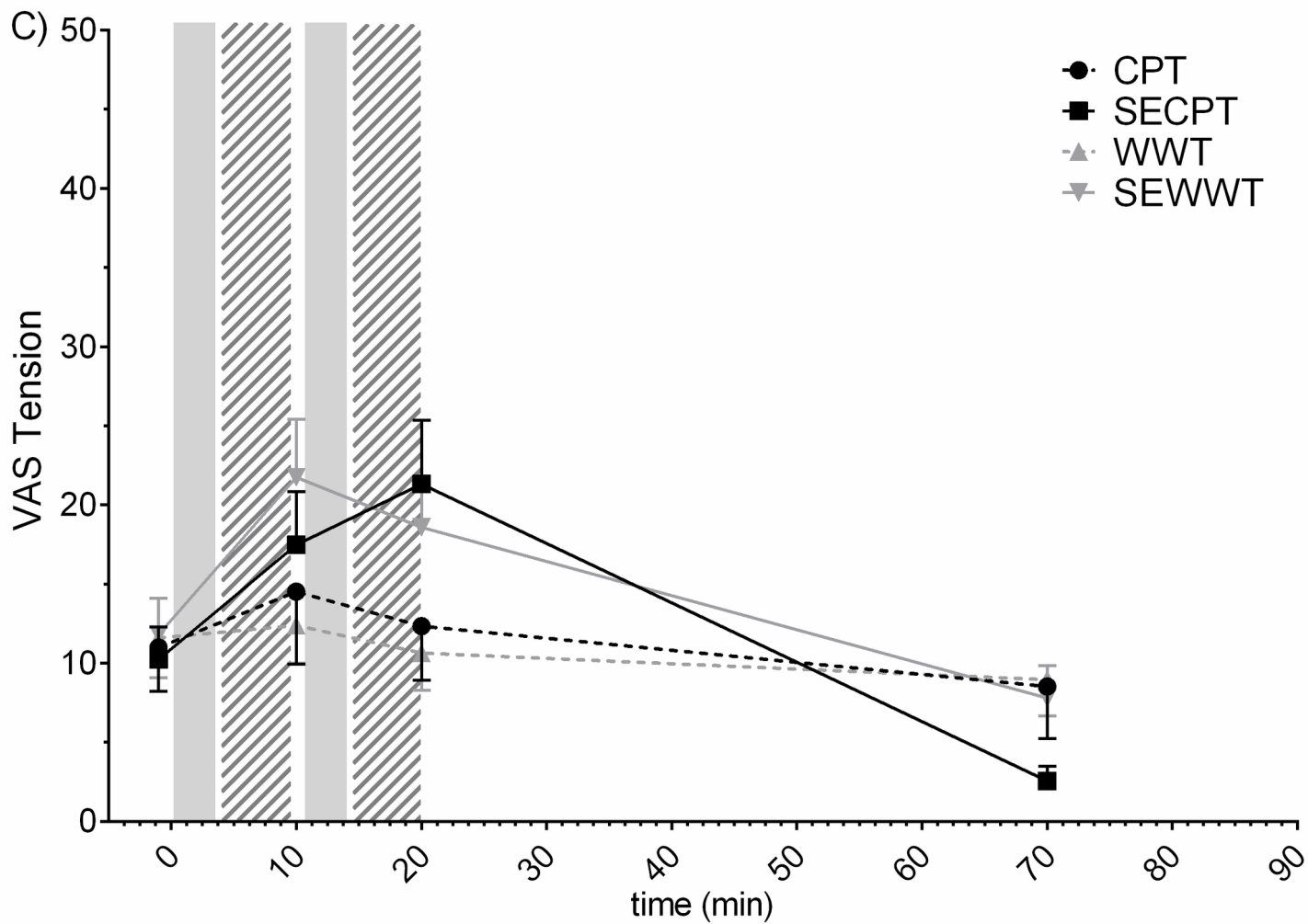

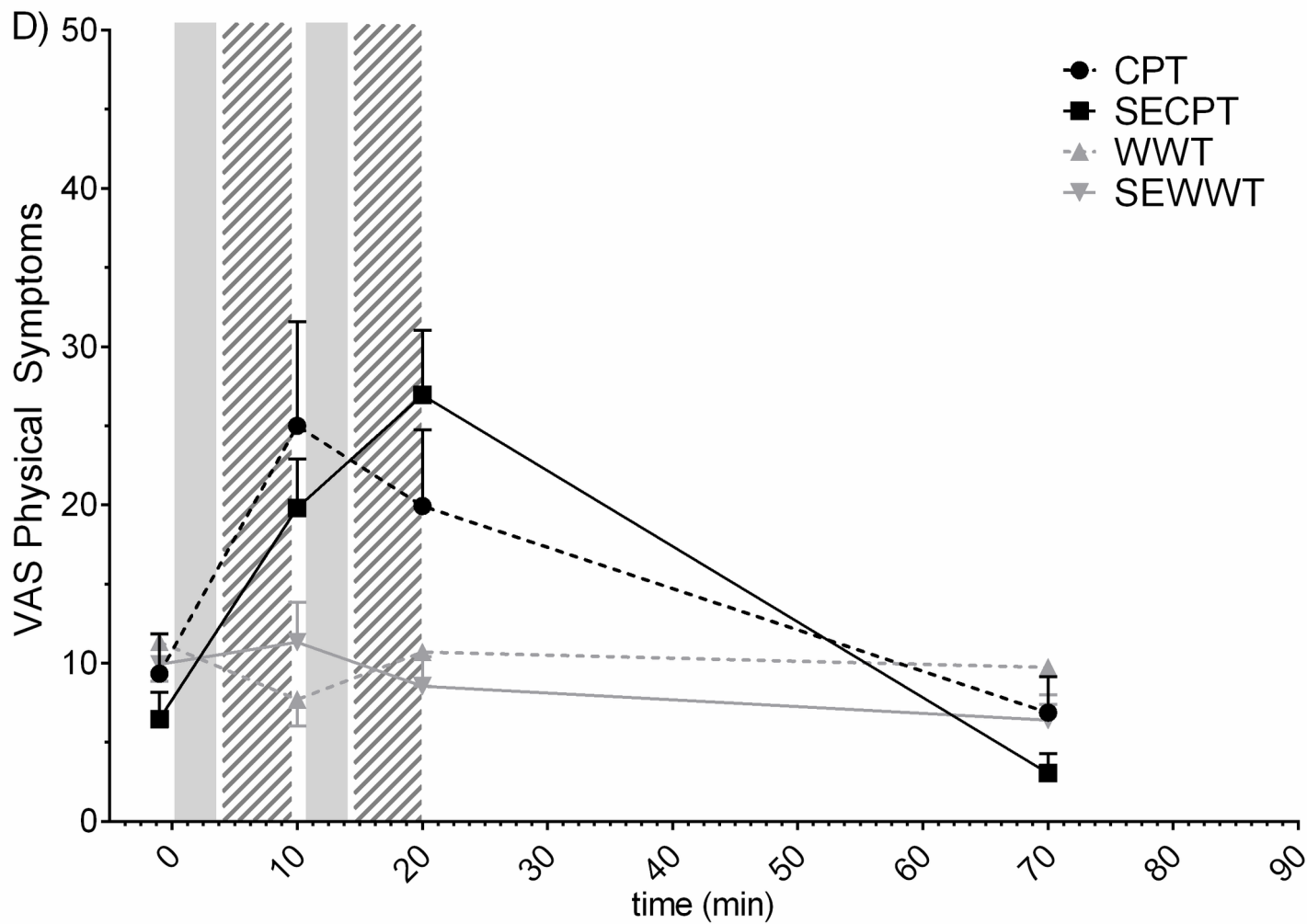

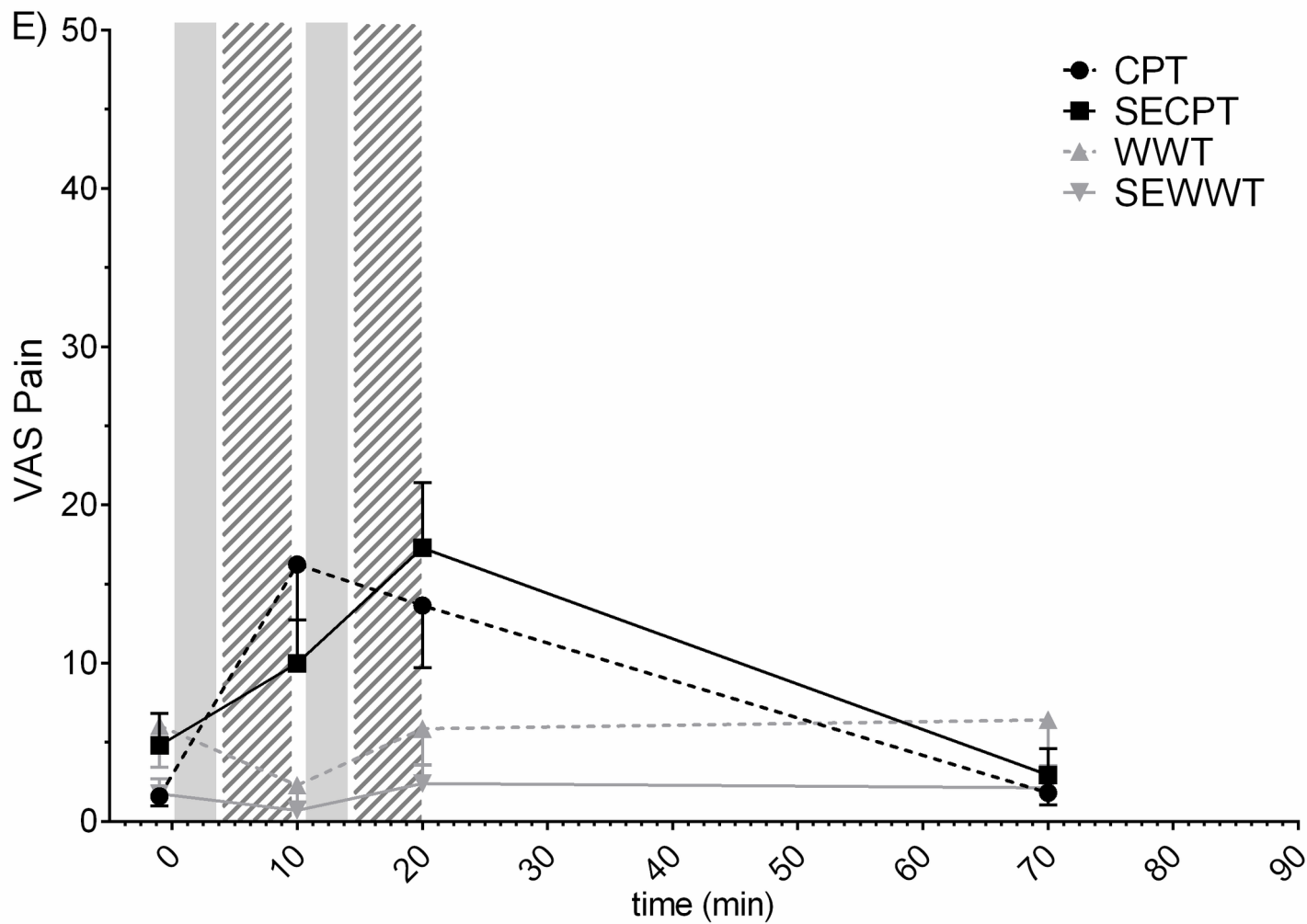

Supplement: S2 Fig — Mean values and standard errors of the mean; solid bars: time of water immersion; shaded bars: decision making; A) stress B) unpleasantness C) tension D) physical symptoms E) pain; WWT = Warm Water Test, SEWWT = Socially Evaluated Warm Water Test, CPT = Cold Pressor Test, SECPT = Socially Evaluated Cold Pressor Test. (PDF) [file pone.0204665.s002.pdf]
