# Supplementary material for: Acute social and physical stress interact to influence social behavior: The role of social anxiety
Source: PLoS One. 2018 Oct 25;13(10):e0204665. doi: 10.1371/journal.pone.0204665 (PMC6201881; doi:10.1371/journal.pone.0204665)
Supplement: S1 Table — F an p values of baseline characteristics. (PDF) [file pone.0204665.s003.pdf]

Table S1. Stastical values of baseline characteristics

	F & p physical stress	*F & p social stress*	*F & p physical stress* social stress*	*F & p social anxiety*	*F & p depressive symptoms*
Cortisol	F(1,95)=0.746 p=0.390	F(1,95)=0.724 p=0.397	F(1,95)=0.153 p=0.696	F(1,95)=1.225 p=0.271	F(1,95)=0.675 p=0.413
Heart Rate during instructions	F(1,85)=0.004 p=0.950	F(1,85)=0.285 p=0.595	F(1,85)=0.330 p=0.567	F(1,85)=0.278 p=0.600	F(1,85)=0.003 p=0.955
Heart Rate baseline	F(1,85)=0.002 p=0.963	F(1,85)=3.168 p=0.079	F(1,85)=0.105 p=0.747	F(1,85)=0.230 p=0.633	F(1,85)=0.001 p=0.981
VAS Stress	F(1,95)=0.567 p=0.453	F(1,95)=0.111 p=0.740	F(1,95)=0.183 p=0.669	F(1,95)=0.210 p=0.648	F(1,95)=0.899 p=0.345
VAS Unpleasantness	F(1,95)=0.120 p=0.730	F(1,95)=0.093 p=0.761	F(1,95)=0.606 p=0.438	F(1,95)=0.667 p=0.416	F(1,95)=1.294 p=0.258
VAS Physical symptoms	F(1,95)=0.937 p=0.335	F(1,95)=0.336 p=0.564	F(1,95)=0.075 p=0.785	F(1,95)=0.148 p=0.701	F(1,95)=2.696 p=0.104
VAS Tension	F(1,95)=0.107 p=0.744	F(1,95)=0.002 p=0.964	F(1,95)=0.030 p=0.864	F(1,95)=0.020 p=0.888	F(1,95)=0.580 p=0.448
VAS Pain	F(1,95)=0.095 p=0.758	F(1,95)=0.005 p=0.942	F(1,95)=2.574 p=0.112	F(1,95)=2.743 p=0.101	F(1,95)=0.413 p=0.522
