# Supplementary material for: Acute social and physical stress interact to influence social behavior: The role of social anxiety
Source: PLoS One. 2018 Oct 25;13(10):e0204665. doi: 10.1371/journal.pone.0204665 (PMC6201881; doi:10.1371/journal.pone.0204665)
Supplement: S2 Table — F an p values of psychological stress response. (PDF) [file pone.0204665.s004.pdf]

Table S2. Stastical values of psychological stress response

	F & p physical stress	*F & p social stress*	*F & p physical stress* social stress*	*F & p social anxiety*	*F & p depressive symptoms*
VAS Stress Increase	$F(1,95)=1.187$ $p=0.279$	$F(1,95)=5.551$ $p=0.021$	$F(1,95)=0.996$ $p=0.321$	$F(1,95)=3.397$ $p=0.068$	$F(1,95)=0.363$ $p=0.548$
VAS Unpleasantness Increase	$F(1,95)=58.414$ $p<0.001$	$F(1,95)=0.642$ $p=0.425$	$F(1,95)=1.998$ $p=0.161$	$F(1,95)=2.553$ $p=0.113$	$F(1,95)=0.424$ $p=0.517$
VAS Physical symptoms Increase	$F(1,95)=28.051$ $p<0.001$	$F(1,95)=0.289$ $p=0.592$	$F(1,95)=0.124$ $p=0.725$	$F(1,95)=0.726$ $p=0.396$	$F(1,95)=0.111$ $p=0.740$
VAS Tension Increase	$F(1,95)=0.539$ $p=0.465$	$F(1,95)=7.901$ $p=0.006$	$F(1,95)=0.001$ $p=0.975$	$F(1,95)=3.591$ $p=0.061$	$F(1,95)=3.875$ $p=0.052$
VAS Pain Increase	$F(1,95)=28.871$ $p<0.001$	$F(1,95)=0.894$ $p=0.347$	$F(1,95)=1.305$ $p=0.256$	$F(1,95)=0.372$ $p=0.543$	$F(1,95)=0.057$ $p=0.812$
