# Supplementary material for: Acute social and physical stress interact to influence social behavior: The role of social anxiety
Source: PLoS One. 2018 Oct 25;13(10):e0204665. doi: 10.1371/journal.pone.0204665 (PMC6201881; doi:10.1371/journal.pone.0204665)
Supplement: S3 Table — F and p values of physiological stress response within subjects results. (PDF) [file pone.0204665.s005.pdf]

Table S3. Stastical values of physiological stress response with repeated measures – Within subject results

	F & p time	*F & p time*physical stress*	*F & p time*social stress*	*F & p Time*physical stress* social stress*	*F & p social anxiety*	*F & p depressive symptoms*
Cortisol	F(2.77, 262.95)=9.520 P<0.001	F(2.77, 262.95)=36.912 p<0.001	F(2.77, 262.95)=0.718 p=0.532	F(2.77, 262.95)=0.545 p=0.638	F(2.77, 262.95)=0.433 p=0.714	F(2.77, 262.95)=0.641 p=0.577
Heart Rate	F(4.96, 421.50)=3.484 P=0.004	F(4.96, 421.50)=8.945 p<0.001	F(4.96, 421.50)=2.852 p=0.015	F(4.96, 421.50)=2.717 p=0.020	F(4.96, 421.50)=0.473 p=0.795	F(4.96, 421.50)=1.176 p=0.320
