# Supplementary material for: Acute social and physical stress interact to influence social behavior: The role of social anxiety
Source: PLoS One. 2018 Oct 25;13(10):e0204665. doi: 10.1371/journal.pone.0204665 (PMC6201881; doi:10.1371/journal.pone.0204665)
Supplement: S4 Table — F and p values of physiological stress response between subjects effects. (PDF) [file pone.0204665.s006.pdf]

Table S4. Stastical values of physiological stress response with repeated measures – Between subjects main effects

	F & p physical stress	*F & p social stress*	*F & p physical stress* social stress*	*F & p social anxiety*	*F & p depressive symptoms*
Cortisol	F(1,95)=17.193 p<0.001	F(1,95)=0.507 p=0.478	F(1,95)=0.128 p=0.721	F(1,95)=0.903 p=0.344	F(1,95)=0.491 p=0.485
Heart Rate	F(1,85)=0.690 p=0.408	F(1,85)=3.893 p=0.052	F(1,85)=0.500 p=0.481	F(1,85)=0.083 p=0.774	F(1,85)=0.183 p=0.711
