# Supplementary material for: Acute social and physical stress interact to influence social behavior: The role of social anxiety
Source: PLoS One. 2018 Oct 25;13(10):e0204665. doi: 10.1371/journal.pone.0204665 (PMC6201881; doi:10.1371/journal.pone.0204665)
Supplement: S5 Table — F values, Wilk'sΛ and p values. (PDF) [file pone.0204665.s007.pdf]

Table S5. Stastical values of physiological stress response – maximum increases of cortisol and heart rate

	F & p physical stress	*F & p social stress*	*F & p physical stress* social stress*	*F & p social anxiety*	*F & p depressive symptoms*
Cortisol Increase	F(1,95)=83.190<br>p<0.001	F(1,95)=0.470<br>p=0.494	F(1,95)=1.169<br>p=0.282	F(1,95)=0.018<br>p=0.894	F(1,95)=0.165<br>p=0.685
MANCOVA HR Increase (multivariate)	F(2,84)=5.997<br>Wilk'sΛ=0.875<br>p=0.004	F(2,84)=1.563<br>Wilk'sΛ=0.964<br>p=0.216	F(2,84)=0.445<br>Wilk'sΛ=0.990<br>p=0.642	F(2,84)=0.202<br>Wilk'sΛ=0.995<br>P=0.818	F(2,84)=0.978<br>Wilk'sΛ=0.977<br>p=0.380
Heart Rate Increase I (between subject)	F(1,85)=11.602<br>p=0.001	F(1,85)=2.540<br>p=0.115	F(1,85)=0.874<br>p=0.353	F(1,85)=0.129<br>p=0.720	F(1,85)=0.516<br>p=0.475
Heart Rate Increase II (between subject)	F(1,85)=2.284<br>p=0.134	F(1,85)=0.125<br>p=0.724	F(1,85)=0.198<br>p=0.658	F(1,85)=0.039<br>p=0.845	F(1,85)=11.602<br>p=0.001
