# Supplementary material for: Acute social and physical stress interact to influence social behavior: The role of social anxiety
Source: PLoS One. 2018 Oct 25;13(10):e0204665. doi: 10.1371/journal.pone.0204665 (PMC6201881; doi:10.1371/journal.pone.0204665)
Supplement: S6 Table — All F and p values. (PDF) [file pone.0204665.s008.pdf]

Table S6. Stastical values of ANCOVA models for behavioral paradigms

	F & p physical stress	*F & p social stress*	*F & p physical stress* social stress*	*F & p social anxiety*	*F & p depressive symptoms*
Trust	F(1,95)=0.631 p=0.429	F(1,95)=0.126 p=0.723	F(1,95)=4.489 p=0.037	F(1,95)=12.522 p=0.001	F(1,95)=0.031 p=0.861
Trustworthiness	F(1,95)=0.205 p=0.652	F(1,95)=1.050 p=0.308	F(1,95)=10.394 p=0.027	F(1,95)=2.269 p=0.135	F(1,95)=0.145 p=0.705
Sharing	F(1,95)=0.390 p=0.534	F(1,95)=3.790 p=0.055	F(1,95)=5.937 p=0.017	F(1,95)=0.112 p=0.738	F(1,95)=0.116 p=0.735
Punishment	F(1,95)=0.505 p=0.479	F(1,95)=0.703 p=0.404	F(1,95)=3.555 p=0.062	F(1,95)=0.267 p=0.607	F(1,95)=0.056 p=0.813
Risk	F(1,95)=0.170 p=0.681	F(1,95)=4.974 p=0.028	F(1,95)=0.200 p=0.656	F(1,95)=0.413 p=0.522	F(1,95)=0.863 p=0.355
