# Supplementary material for: Acute social and physical stress interact to influence social behavior: The role of social anxiety
Source: PLoS One. 2018 Oct 25;13(10):e0204665. doi: 10.1371/journal.pone.0204665 (PMC6201881; doi:10.1371/journal.pone.0204665)
Supplement: S7 Table — All parameters of significant models. (PDF) [file pone.0204665.s009.pdf]

Table S7. Stepwise regression to explore relationships between of stress systems and trust

			Trust			
condition	model	R^2	R^2_{adj}	p	predictor	β
WWT	n.s.					
SEWWT	1	0.223	0.194	0.010	VAS Unpleasantness Increase	0.033
CPT	1	0.505	0.470	0.002	VAS Unpleasantness Increase	0.037
SECPT	1	0.364	0.324	0.008	Cort Increase	-0.116
	2	0.598	0.545	0.001	Cort Increase<br>VAS Stress Increase	-0.153<br>-0.048
