# Supplementary material for: Acute social and physical stress interact to influence social behavior: The role of social anxiety
Source: PLoS One. 2018 Oct 25;13(10):e0204665. doi: 10.1371/journal.pone.0204665 (PMC6201881; doi:10.1371/journal.pone.0204665)
Supplement: S8 Table — All parameters of significant models. (PDF) [file pone.0204665.s010.pdf]

Table S8. Stepwise regression to explore relationships between of stress systems and trustworthiness

Trustworthiness						
condition	model	R^2	R^2_{adj}	p	predictor	β
WWT	n.s.					
SEWWT	n.s.					
CPT	n.s.					
SECPT	1	0.375	0.336	0.007	Cort Increase	-0.122
