# Supplementary material for: Acute social and physical stress interact to influence social behavior: The role of social anxiety
Source: PLoS One. 2018 Oct 25;13(10):e0204665. doi: 10.1371/journal.pone.0204665 (PMC6201881; doi:10.1371/journal.pone.0204665)
Supplement: S9 Table — All parameters of significant models. (PDF) [file pone.0204665.s011.pdf]

**Table S9. Stepwise regression to explore relationships between of stress systems and sharing**

| | | | Sharing | | | |
|---|---|---|---|---|---|---|
| condition | model | $R^2$ | $R^2_{adj}$ | p | predictor | β |
| WWT | n.s. | | | | | |
| SEWWT | n.s. | | | | | |
| CPT | 1 | 0.253 | 0.199 | 0.047 | Heart Rate Increase | -0.221 |
| SECPT | 1 | 0.245 | 0.198 | 0.037 | Cort Increase | -0.112 |
