# Supplementary material for: Acute social and physical stress interact to influence social behavior: The role of social anxiety
Source: PLoS One. 2018 Oct 25;13(10):e0204665. doi: 10.1371/journal.pone.0204665 (PMC6201881; doi:10.1371/journal.pone.0204665)
Supplement: S10 Table — All parameters of significant models. (PDF) [file pone.0204665.s012.pdf]

Table S10. Stepwise regression to explore relationships between of stress systems and punishment

			Punishment			
condition	model	R^2	R^2_{adj}	p	predictor	β
WWT	1	0.150	0.117	0.042	Heart Rate Increase	-0.136
SEWWT	n.s.					
CPT	n.s.					
SECPT	n.s.					
